# Pharmacological mechanisms and therapeutic prospects of plant metabolites from traditional Chinese medicine in regulating programmed cell death in Alzheimer’s disease

**DOI:** 10.3389/fphar.2025.1605214

**Published:** 2025-09-24

**Authors:** Pengyu Pan, Tengyu Zhao, Jian Zhang, Yuhan Zhou, Xinyue Zhang, Quan Li, Yanyan Zhou

**Affiliations:** ^1^ School of Basic Medicine, Heilongjiang University of Chinese Medicine, Harbin, China; ^2^ Key Laboratory of Basic Theory Research on Traditional Chinese Medicine, Harbin, Heilongjiang, China

**Keywords:** Alzheimer’s disease, traditional Chinese medicine, programmed cell death, plant metabolites, neuroprotection, ferroptosis, Apoptosis

## Abstract

Alzheimer’s disease (AD) is a progressive neurodegenerative disorder marked by cognitive decline and neuronal loss. Multiple forms of programmed cell death (PCD)—including apoptosis, pyroptosis, ferroptosis, cuproptosis, and disulfidoptosis—contribute to its pathogenesis, regulated by protein families such as caspases, RIPKs, gasdermins, and ATGs. Plant metabolites widely distributed across medicinal plants and enriched in botanical drugs used in traditional Chinese medicine (TCM), such as alkaloids, flavonoids, saponins, and polysaccharides, have attracted increasing attention for their potential regulatory effects on these PCD pathways. These metabolites are not unique to TCM, but their prevalence in TCM prescriptions provides a valuable framework for pharmacological investigation. Their biological activities are often determined by structural features—for example, the isoquinoline scaffold of berberine enhances membrane permeability, facilitating neuroprotective actions. Despite substantial research, comprehensive summaries remain limited. This review systematically integrates progress from the past 2 decades on how plant metabolites, particularly those enriched in TCM botanical drugs, regulate PCD in AD, with the aim of clarifying pharmacological mechanisms and highlighting prospects for drug discovery and clinical translation.

## 1 Introduction

Alzheimer’s disease (AD) is a neurodegenerative disease with insidious onset, characterized by progressive cognitive decline and neuronal loss. Epidemiological data show that the incidence of AD is positively correlated with age. Currently, Currently, over 50 million people worldwide are affected, and approximately one-tenth of individuals older than 65 years develop the disease (Luo et al., 2023; Hou et al., 2019). AD has become one of the most expensive, deadly and burdensome diseases and a major challenge for global public health. Although existing drugs such as acetylcholinesterase inhibitors can temporarily relieve symptoms, they cannot reverse the disease process and have significant side effects. Therefore, it is essential to explore novel multi-target therapeutic strategies (Sharma et al., 2024).

Cell death can be broadly classified into two types: accidental cell death (ACD) and programmed cell death (PCD). ACD occurs when external insults overwhelm the cell’s repair capacity, leading to necrosis. In contrast, PCD is a regulated process critical for removing damaged cells, promoting tissue renewal, supporting organismal development, defending against pathogens, and maintaining homeostasis. Depending on the context, PCD can be induced, inhibited, or modulated. Over the past five decades, several distinct PCD pathways have been identified, including apoptosis, necroptosis, autophagy, pyroptosis, ferroptosis, cuproptosis, and disulfidoptosis (Fricker et al., 2018).

In recent years, research on plant metabolites used in traditional Chinese medicine (TCM) has revealed their potential therapeutic advantages in AD (Siddiqui et al., 2024a; Siddiqui et al., 2024b). These metabolites exert multi-target and multi-pathway regulatory effects, enabling more precise therapeutic strategies. For example, alkaloids can modulate apoptosis, ferroptosis, and autophagy in neuronal cells; saponins can regulate apoptosis, pyroptosis, and ferroptosis; and flavonoids influence apoptosis, pyroptosis, autophagy, and ferroptosis simultaneously. Notably, these pathways exhibit extensive crosstalk, with autophagy serving as a central hub. However, the exact roles of plant metabolites in regulating PCD and the underlying molecular mechanisms remain incompletely understood. Importantly, this review emphasize that the pharmacological effects of these metabolites are grounded in structure–activity relationships (SAR). All effects are supported by experimental data at defined doses rather than general claims of efficacy. For instance, salidroside demonstrates optimal effects at 40 μM in HT22 cells and 50 mg/kg day in Aβ1–42-induced AD mice, whereas berberine shows protective effects at 30–60 μM in ferroptosis models. Incorporating representative dose ranges ensures scientific rigor and prevents overstating the uniqueness of TCM-derived metabolites.

**FIGURE 1:**
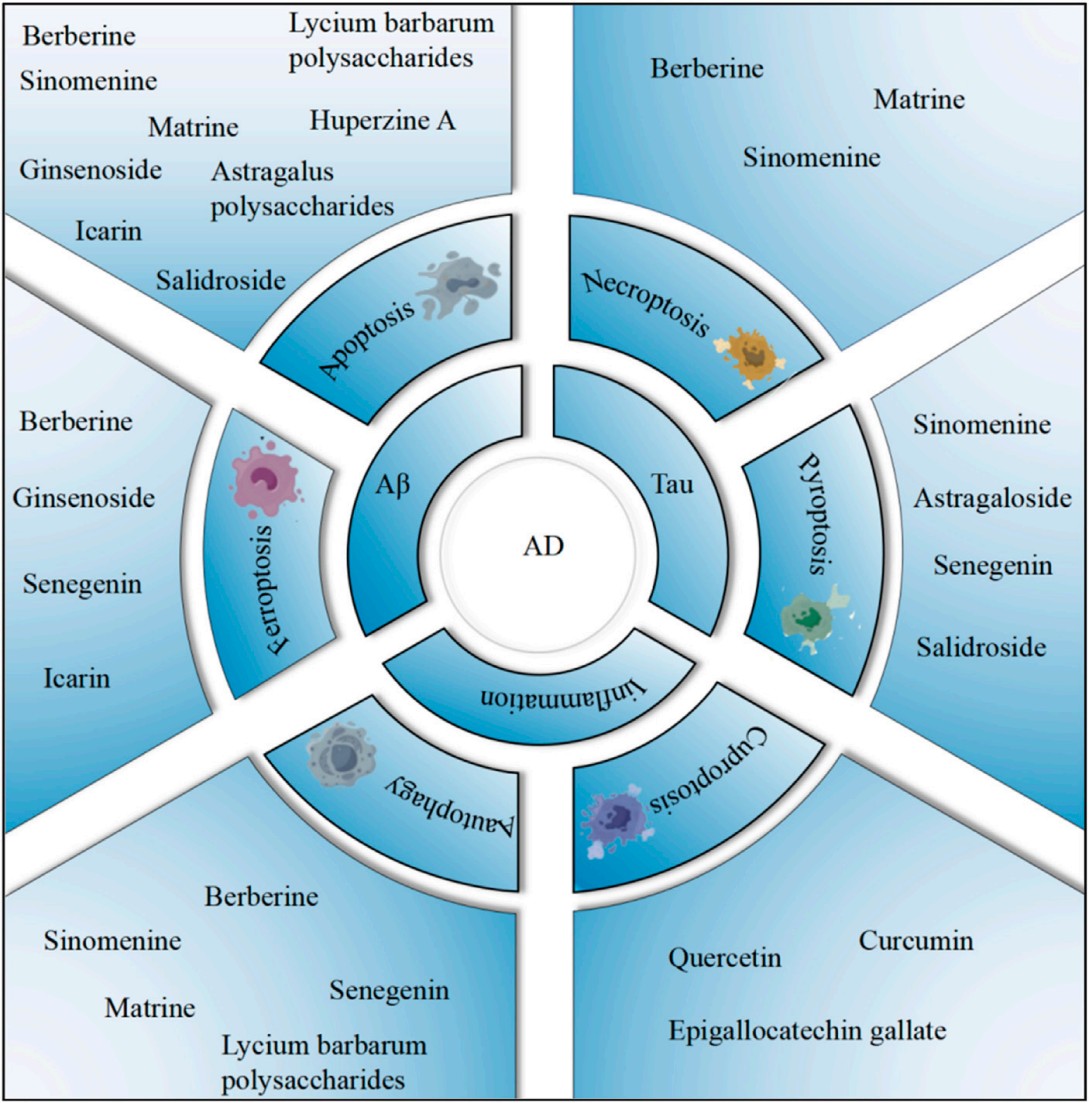
The relationship between PCD and AD.

In this review, we aim to elucidate the mechanisms by which plant metabolites associated with TCM regulate PCD in AD. These insights may not only reveal novel therapeutic targets for AD prevention and treatment but also provide a foundation for future experimental and clinical studies (Figure 1).

## 2 Forms of PCD

PCD is a form of cell death in multicellular organisms regulated by a variety of signaling pathways, which plays a key role (Zhang L. et al., 2024) in body development, tissue remodeling, and disease prevention and control. Under normal physiological conditions, PCD plays an indispensable role as an important protective mechanism to maintain cell homeostasis (Yang K. et al., 2022). Under pathological conditions, abnormal PCD levels, whether insufficient or excessive, may lead to serious consequencessuch as neurodegenerative diseases, autoimmune diseases and cancer (Bello et al., 2018; Zhang Q. et al., 2022). Each PCD mode has its own unique morphological characteristics, biochemical pathways and regulatory mechanisms.

### 2.1 Apoptosis

**FIGURE 2:**
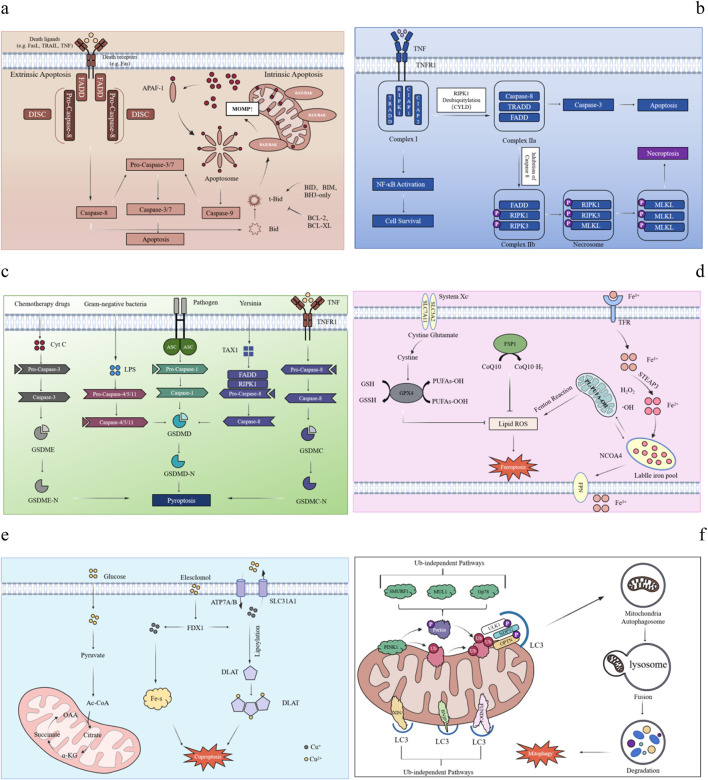
Mechanisms of various PCD. Mechanism diagram of PCD in different forms. **(a)** Apoptosis: The endogenous and exogenous apoptosis pathways and their cross-regulation. In the endogenous pathway, ligand binding to a cell surface receptor recruits FADD to form DISC, activating Caspase-8 and cleaving Caspase-3/7. In the exogenous pathway, pro-apoptotic proteins (Bax, Bak) trigger Cytc release from mitochondria, forming apoptosome with Apaf-1, activating Caspase-9 and cleaving Caspase-3/7. Cross-pathway regulation occurs when Caspase-8 cleaves Bid into tBid, enhancing mitochondrial signaling. **(b) **Necroptosis: Taking the binding of TNF-α to TNFR1 as an example, activation of TNFR1 recruits TRADD, RIPK1, and cIAP1/2 to form complex **(I)**. If caspase-8 is inhibited, complex I dissociates, RIPK1 combines with RIPK3 to form the necrosome (complex IIb), and phosphorylation of RIPK1, RIPK3, and MLKL disrupts membrane integrity, triggering necroptosis. **(c)** Pyroptosis: Pathogen-associated molecular patterns (PAMPs) are recognized by pattern recognition receptors (PRRs), which recruit the adaptor protein ASC. This activates Caspase-1 (classical pathway) or Caspase-4/5/11 (non-classical pathway). The activated Caspases cleave Gasdermin D (GSDMD), forming pores in the cell membrane. These pores disrupt osmotic balance, causing pyroptosis and releasing inflammatory mediators. **(d)** Ferroptosis: The inhibition of System Xc^−^ reduces cystine uptake, depletes intracellular cysteine, and blocks glutathione (GSH) synthesis, preventing lipid peroxide reduction and causing peroxide accumulation. Increased intracellular free Fe^2+^ generates ROS *via* the Fenton reaction, promoting lipid peroxidation. The FSP1-CoQ10 antioxidant axis, independent of the GPX4 pathway, eliminates lipid radicals; its dysfunction triggers ferroptosis. **(e)** Cuproptosis: Cu^2+^ accumulates excessively in cells dependent on mitochondrial respiration, it binds to lipoylated DLAT, inducing abnormal oligomerization of DLAT. This increase in insoluble DLAT levels can result in cytotoxicity and subsequently lead to cell death. Conversely, FDX1 facilitates the reduction of Cu^2+^ to the more toxic Cu^+^, which inhibits Fe-S cluster protein synthesis and ultimately induces apoptosis. **(f)** Autophagy: Loss of mitochondrial membrane potential (ΔΨm) causes PINK1 accumulation on the outer membrane of damaged mitochondria, activating Parkin *via* ubiquitin phosphorylation and recruiting it to the mitochondrial surface. Autophagy occurs through two main pathways: the ubiquitin-dependent pathway, where autophagy receptors like OPTN and NDP52 bind LC3, and the direct labeling pathway, where receptors like BNIP3, NIX, and FUNDC1 directly interact with LC3.

Apoptosis is a form of PCD in multicellular organisms. The process can be divided into two main stages: the formation of apoptotic bodies and their subsequent recognition, phagocytosis and degradation by neighboring cells. The signal transduction mechanism of apoptosis mainly includes the triggeringof endogenous and exogenous pathways (Elliott and Ravichandran, 2010; Galluzzi et al., 2018). When DNA damage, hypoxia and metabolic stress occur, the endogenous pathway of apoptosis, also known as mitochondrial pathway, will be triggered by the decrease of mitochondrial membrane potential and the increase of membrane permeability (Nossing and Ryan, 2023). Bcl-2 family members, including anti-apoptotic proteins such as Bcl-2 and Bcl-xl, pro-apoptotic proteins such as Bax and Bak, and BH3-only proteins such as Bad and Bid, are key mediators in regulating mitochondrial membrane permeability (Czabotar et al., 2014). Under the action of various pro-apoptotic signals, pro-apoptotic proteins such as Bax and Bak are no longer inhibited, and extend to the mitochondrial outer membrane and promote mitochondrial outer membrane permeability (MOMP), leading to the increase of mitochondrial membrane permeability (Wei et al., 2001; Tait and Green, 2010; Bock and Tait, 2020). Cytochrome C (Cytc) is transferred from the mitochondria to the cytoplasm and binds to apoptosis protease-activating factor-1 (Apaf-1) to form a multimercomplex to activate Pro-Caspase 9, which then activates downstream apoptotic effector proteins to lead to apoptosis (Cain et al., 2002). When the extracellular environment is disturbed, death receptors on the membrane surface receive the death signal transmitted by extracellular ligands and activate the extrinsic apoptotic pathway (Tenev et al., 2011). There are at least eight apoptosis-related death receptor protein family members on the surface of mammalian cells, among which Fas (APO-1/CD95), TNFR1, TRAILR1 (DR4) and TRAILR2 (DR5) are the most common (Suliman et al., 2001; Peter and Krammer, 1998; Ashkenazi and Dixit, 1998). The cytoplasmic portion of these receptors contains the Death Domain (DD), which is used to recruit key molecules (Han Y. H. et al., 2023). After binding to their ligands, the receptors form death-inducing signaling complex (DISC) to initiate the extrinsic apoptotic pathway (Yang et al., 2024). Fas and TNFR1 are typical representatives. Upon binding to FasL and TNF, respectively, it triggers trimerization of intracellular receptors and exposure of death domains to recruit adaptor proteins FADD and TRADD (Macewan, 2002; Guicciardi and Gores, 2009). FADD recruits and activates the precursor Caspase-8, which converts to the apoptosis-initiating protein Caspase-8. Subsequently, the activated Caspase-8 activates the apoptotic effector protein Caspase-3, leading to PCD (Dickens et al., 2012). In contrast, TRADD activates TNFR-related factor TRAF-2 upon binding to RIP1. TRAF-2 interacts with TRAF-1 to recruit cIAPs to form a complex that inhibits Caspase-8 activity and its release to prevent apoptosis and promote survival (Hsu et al., 1996a; Hsu et al., 1996b). In addition, the endogenous and exogenous pathways of apoptosis are not completely independent, but can interact together to regulate the apoptotic process under specific conditions. For example, activated Caspase-8 can cleave Bid to produce t-Bid, which is transported to mitochondria to promote the release of Cytc and ultimately induce apoptosis (Zhang et al., 2021; Perez and White, 2000) (Figure 2a).

### 2.2 Necroptosis

Necroptosis is a form of PCD between apoptosis and necrosis. Since the concept of PCD was proposed in the 20th century, apoptosis has been regarded as the main form of PCD, and it is generally believed that it is closely related to the activation of Caspase family (Grootjans et al., 2017). However, with the development of research, scientists have found that death receptor-induced cell death still occurs even when Caspase family is inhibited. This mode of death shows typical necrosis-like morphological features such as cell swelling and deformation, cell membrane rupture. This suggests that Caspase activation is not the only mechanism that determines cell fate during PCD and that there are other possible cell death pathways (Weinlich et al., 2017; Galluzzi et al., 2017). This novel mode of cell death, necroptosis, was first reported by Degterev et al., in 2005 (Degterev et al., 2005). The activation of necroptosis is an active and dependent process of intracellular signal transduction, and its regulatory mechanism is similar to that of apoptosis. Among them, TNF-α is the most important upstream signaling molecule of necroptosis, and its receptor TNFR1 can recruit a variety of proteins, including TRADD, RIPK1, CIAP1, CIAP2 and LUBAC, which together form a signaling complex named “complex Ⅰ” (Annibaldi and Meier, 2018). In most cases, CIAP and LUBAC in complex I promote the ubiquitination of RIPK1 through K63 linkage and linear ubiquitin chains, which further recruit TAK and IKK complexes to activate NF-κB and MAPK signaling pathways, thereby promoting the production of inflammatory factors and maintaining cell survival (Annibaldi and Meier, 2018). In addition, the K63-linked ubiquitin chain on RIPK1 is hydrolyzed under the action of two deubiquitinating enzymes, CYLD and A20, thereby promoting the formation of complex II (Priem et al., 2020; Lork et al., 2017; Chen, 2012). The dissociated RIPK1 forms complex Ⅱb together with FADD and Caspase-8. When Caspase-8 activity is inhibited, RIPK1 further recruits and phosphorylates RIPK3 to form a RIPK1-RIPK3 necrosome (Li et al., 2012; Cho et al., 2009). RIPK1 and RIPK3 activate MLKL through autophosphorylation and cross-phosphorylation *via* homotypic interaction in the RHIM domain. Subsequently, RIPK3 catalyzes the phosphorylation of MLKL at Thr357 and Ser358 in its pseudokinase domain. After MLKL phosphorylation, its conformation changes, exposing the four-helix bundle (4HB) domain, which promotes MLKL oligomerization (Murphy et al., 2013). Oligomerization of MLKL migrates to the plasma membrane, where it forms a permeable pore, destroying the integrity of the membrane, and eventually causing programmed necrosis (Chen et al., 2014; Wang et al., 2014; Kaczmarek et al., 2013). In addition to TNF, other stimulating factors such as Fas, DR3, DR4, DR5 and DR6 can also trigger this process (Bi et al., 2017; Strilic et al., 2016) (Figure 2b).

### 2.3 Pyroptosis

Pyroptosis, also known as inflammatory necrosis of cells, is characterized by the continuous expansion of cells until cell membrane rupture, resulting in the release of cell contents and triggering an intense inflammatory response. Pyroptosis is an important part of the body’s innate immune response and plays a key role in the pathological process of immune system and nervous system diseases. The process is usually induced and executed by members of the Gasdermin (GSDM) protein family. The GSDM family of proteins contains six members, Five of them are Gasdermin A (GSDMA), Gasdermin B (GSDMB), Gasdermin C (GSDMC), Gasdermin D (GSDMD) and Gasdermin E (GSDME) -- each plays a unique rolein pore formation and pyroptosis-induced cell death (Yang F. et al., 2022). The GSDM-mediated pyroptosis pathway can proceed in either an inflammasome-dependent or an inflammasome-independent manner. The canonical inflammasome pathway is mediated by Caspase-1, while the non-canonical inflammasome pathway is mediated by Caspase-11 (mouse) and Caspase-4/5 (human), respectively. Both pathways eventually lead to the cleavage of GSDMD to generate its N-terminal and C-terminal fragments, of which the N-terminal fragment (GSDMD-N, or pore-forming domain PFD) is further oligomerized to form holes in the cell membrane (Toldo and Abbate, 2024). In the classical inflammasome pathway, GSDMD cleavage activates Pro-IL-1β and Pro-IL-18 to form mature inflammatory factors IL-1β and IL-18, which are released to the outside of the cell, followed by pyroptosis and lytic death of the cell (Rathinam and Fitzgerald, 2016; Broz and Dixit, 2016). In the non-canonical pathway, when stimulated by Gram-negative bacteria and their lipopolysaccharide (LPS), Caspase-4, Caspase-5 and Caspase-11 can directly bind to and be activated by LPS to cleave GSDMD protein. The N-terminus of GSDMD protein can not only mediate cell membrane lysis and pyroptosis, but also activate NLRP3 inflammasome to activate Caspase-1, and finally produce and release IL-1β (Hagar et al., 2013; Shi et al., 2014). In addition to the above two modes of pyroptosis, Caspase-3/8-mediated pathway and GSDM protein-related induction pathway can also trigger pyroptosis (Toldo and Abbate, 2024). Specifically, Caspase-3 can be activated by a variety of stimulants such as drugs and viruses, and specifically cleave GSDME protein to mediate the pyroptosis process (Liu et al., 2022). However, the mechanism of caspase-8-mediated pyroptosis is more complex: first, after the tumor necrosis factor (TNF) -mediated death receptor signaling is activated, GSDMC can be cleaved by Caspase-8, and then apoptosis converts to pyroptosis in GSDMc-expressing cells (Qin et al., 2025). Second, when YopJ, a *Yersinia* effector protein, inhibited the activity of TGF-β-activated kinase-1 (TAK1) or IKK kinase, it activated the RIPK1-Caspase-8 pathway, leading to the cleavage of GSDMD and accelerated the occurrence of pyroptosis (Orning et al., 2018; Sarhan et al., 2018). Studies have shown that GSDM mediated pyroptosis plays an important role in pathological processes such as neuroinflammation and mental diseases. As a defense mechanism, pyroptosis not only helps to remove pathogens and restore the stability of the intracellular environment, but also enhances the recognition and clearance efficiency of phagocytes such as neutrophils by encapsulating toxic substances. However, if the regulation is not balanced, it may lead to excessive tissue inflammation, which is closely related to a variety of nervous system diseases such as AD and Parkinson’s disease (Zhang S. et al., 2022) (Figure 2c).

### 2.4 Ferroptosis

Ferroptosis is a non-apoptotic cell death mode dependent on iron, which was first proposed by Dixon et al., in 2012 (Dixon et al., 2012). As an essential trace element for cells to maintain their morphology and function, iron plays an important role in oxygen transport, ATP synthesis, and DNA biosynthesis. Under normal physiological conditions, divalent iron (Fe^2+^) from food is transported to intestinal epithelial cells for absorption through divalent metal transporter 1 (DMT1). The absorbed Fe^2+^ is oxidized to Fe^3+^ in the intestinal mucosal epithelial cells, which is bound to transferrin (TF) and transported to the cells (Outten and Theil, 2009). The transferrin receptor (TFR1) on the cell surface recognizes and binds to a complex carrying trivalent iron (Tf-Fe^3+^) (Frazer and Anderson, 2014). It endocytoses into the cell interior. Once inside the cell, prostate transmembrane epithelial 3 antigen (STEAP3) reduces Fe^3+^ to Fe^2+^. Some Fe^2+^ is stored in the available LIP, while excess Fe^2+^ is stored in ferritin (Bogdan et al., 2016). Ferritin is an important iron storage protein, which can dynamically regulate the oxidation of ferrous ions (Fe^2+^) to ferric iron (Fe^3+^) for intracellular enzymatic reactions or storage. When the Fe^2+^ stored in the LIP binds to nuclear receptor coactivator 4 (NCOA4), it will trigger ferroautophagy and form autolysosome, leading to the degradation of ferritin and the release of large amounts of Fe^2+^ to complete the iron metabolism process (Hou et al., 2016). However, excessive iron uptake, decreased iron storage, or blocked iron removal due to a variety of factors may lead to intracellular Fe^2+^ overload. Excess Fe^2+^ undergoes the Fenton reaction with hydrogen peroxide (H_2_O_2_) to generate strongly oxidizing hydroxyl radicals (·OH). These free radicals can directly attack polyunsaturated fatty acids (such as arachidonic acid AA and adrenal acid AdA) in the cell membrane, trigger lipid peroxidation, accelerate the occurrence of lipid peroxidation, produce a large number of reactive oxygen species (ROS), and then promote ferroptosis (Chen et al., 2023). Lipid peroxidation triggered by iron metabolism disorder is a key mechanism leading to cell damage and ferroptosis. In addition, the imbalance of the antioxidant system of amino acids can also trigger the accumulation of lipid peroxides, which in turn promotes the occurrence of ferroptosis. A variety of antioxidant pathways have been evolved to resist oxidative stress damage, including cystine glutamate antitransporter receptor (system XC^−^) pathway, glutathione peroxidase 4 (GPX4) pathway, inhibitor of ferroptosis protein 1-Coenzyme Q10 (FSP1-CoQ10) pathway and P53 pathway (Zheng and Conrad, 2020; Bersuker et al., 2019). System XC^−^ is an important amino acid transport system, which consists of a heterodimer of solute transport family 7A11 (SLC7A11) and SLC3A2 subunits. The main function of this system is to transport cystine, the precursor of the major intracellular antioxidant glutathione (GSH), into the cell. During ferroptosis, GSH reduces lipid peroxides to normal lipids through GPX4 to prevent their accumulation, thereby inhibiting the occurrence of ferroptosis. When system XC^−^ is inhibited or its function is reduced, the intracellular cystine uptake is reduced, resulting in decreased GSH synthesis, which in turn reduces the antioxidant capacity of cells, increases the accumulation of lipid peroxides, and ultimately promotes ferroptosis (Jiang et al., 2015). P53 can inhibit the uptake of cystine by system XC- by down-regulating the expression of cystine, affect the activity of GPX4, reduce the antioxidant capacity of cells, and accelerate the occurrence (Xie et al., 2017) of ferroptosis. Coenzyme Q10 (CoQ10), as an important lipid-soluble antioxidant *in vivo*, can effectively capture free radicals generated during lipid peroxidation and inhibit the accumulation of lipid peroxides. In addition, CoQ10 is able to regenerate other antioxidants such as α-tocopherol (vitamin E), thereby indirectly inhibiting lipid peroxidation. Ferroptosis inhibitor protein 1 (FSP1) is a key protein in the regulation of ferroptosis. It catalyzes the REDOX cycle of CoQ10 to generate ubiquinol, which further reduces the accumulation of lipid peroxides and prevents the occurrenceof ferroptosis (Bersuker et al., 2019; Kuang et al., 2020) (Figure 2d).

### 2.5 Cuproptosis

Copper is an indispensable trace element in the physiological processes of all types of cells in the human body. It is widely involved in mitochondrial respiration, antioxidant reactions, biosynthesis of biological compounds, and energy metabolism. Therefore, the uptake, distribution and metabolism of copper are strictly regulated (Tang et al., 2022; Maung et al., 2021). The imbalance of copper homeostasis in the body will trigger a form of cell death caused by excessive copper, namely, Cuproptosis (Tsvetkov et al., 2022). The basic metabolic process of copper relies on three key copper transporters: SLC31A1 is responsible for copper uptake, while ATP7A and ATP7B are responsible for copper efflux. Prostate six-transmembrane epidermal antigen (STEAP), a metalloreductase located on the surface of small intestinal epithelial cells, reduces Cu^2+^ to Cu^+^ and subsequently enters the cell through the high-affinity transmembrane transporter copper transporter 1 (CTR1). Once inside the cell, Cu^+^ binds to copper chaperones through the cytoplasmic, mitochondrial and Golgi pathways, and is targeted for delivery to different copper proteins (Galler et al., 2020). For example, in the cytoplasm, superoxide dismutase copper chaperones (CCS) activate superoxide dismutase 1 (SOD1), which plays an important antioxidant role by regulating the distribution of SOD1 in the cell, ensuring the balance of ROS in the body, and preventing oxidative damage caused by copper overload (Wang et al., 2024). In mitochondria, Cytc oxidase copper chaperone 17 (Cox17) mediates the transport of copper from the cytoplasm to the mitochondria and participates in the respiratory chain and REDOX pathway. In addition, most of the Cu^+^ entering hepatocytes is transported to the trans-Golgi network (TGN) through the antioxidant protein 1 (ATOX1), and copper is excreted by the major transporter ATP7A/B. Together, they maintain the body’s copper homeostasis (Da et al., 2022). When copper homeostasis is disrupted for various reasons, the intracellular copper concentration increases. Copper ionophores such as elesclomol (ES) and disulfiram (DSF) can directly bind to extracellular Cu^2+^ and actively enter the cell, where they are targeted and enriched inmitochondria through the mitochondrial membrane (Tsvetkov et al., 2022; Lutsenko et al., 2010). On the one hand, Cu^2+^ is reduced to more toxic Cu^+^ under the action of ferredoxin 1 (FDX1), which inhibits the synthesis of Fe-S Cluster proteins related to mitochondrial respiration and triggers a proteotoxic stress response that eventually leads to cell death (Tsvetkov et al., 2019). On the other hand, FDX1, as an upstream regulator of protein lipoacylation, participates in the tricarboxylic acid cycle and binds to lipoacylated mitochondrial proteins (such as DLAT), causing protein oligomerization and producing ROS to induce oxidative stress. These abnormal processes together eventually lead to cuproptosis of cells (Sun et al., 2023). Therefore, the accumulation of intracellular copper exceeding the limit is the direct cause of cuproptosis. Cu death can be induced by directly delivering Cu^2+^ into cells using copper ionophore, over-expressing the copper transporter SLC31A1/CTR1, or down-regulating the expression of ATP7A/B protein to increase the intracellular Cu+ concentration (Xie J. et al., 2023) (Figure 2e).

### 2.6 Autophagy

Autophagy is a process of engulfing damaged or aged organelles and proteins, encapsulating them into vesicles to form autolysosomes for degradation, and ultimately achieving cell self-renewal. This process can be divided into two types: selective and non-selective (Gatica et al., 2018; Nakatogawa, 2020). In view of the important role of selective autophagy in maintaining body homeostasis and its ability to target the removal of damaged mitochondria, mitophagy, as an important branch of selective autophagy, has become a hot research field (Vargas et al., 2023). In mammals, the regulatory pathways of mitophagy can be classified into two categories (Khaminets et al., 2016): Ubiquitin-dependent and independent.

PINK1/Parkin signaling, which is dependent on extensive ubiquitylation of mitochondrial surface proteins, is the classical pathway to mediate mitophagy. Pten-induced kinase 1 (PINK1) plays a key role in this process (Harper et al., 2018). Under normal physiological conditions, PINK1 is transported to the inner mitochondrial membrane through the mitochondrial outer membrane translocase complex (TOM) and is rapidly degraded to maintain mitochondrial quality control (Bausewein et al., 2020; Ruth et al., 2014; Sekine et al., 2019). When Mitochondrial damage results in loss of Mitochondrial Membrane Potential (MMP), PINK1 is unable to enter the inner Membrane and accumulates steadily on the Outer Mitochondrial Membrane (OMM). It undergoes rapid autophosphorylation and undergoes a series of modifications on Parkin, such as binding to Ser 65 phosphorylated ubiquitin (pSer 65-Ub) to activate E3 ubiquitin ligase activity. In addition, PINK1 interacts with microtubule-associated protein 1A/1B light chain 3 (LC3) to initiate autophagosome formation and fusion with lysosomes, ultimately promoting the degradationof damaged mitochondria (Koyano et al., 2014; Rasool et al., 2018).

In addition to participating in ubiquitin-dependent mitophagy, some mitochondrial proteins also act as mitophagy receptors, directly mediating the targeted binding and degradation process of dysfunctional mitochondria to autophagosomes (Palikaras et al., 2018). For example, autophagy receptor proteins such as BNIP3, NIX/BNIP3L, FUNDC1 and AMBRA1 interact with LC3 and GABARAP through their LIR domains to efficiently mediate mitochondrial clearance (Gatica et al., 2018; Marinkovic et al., 2021; Hanna et al., 2012) (Figure 2f) (Table 1).

**TABLE 1:** The biological differences of PCD patterns.

Characteristics		Apoptosis	Necroptosis	Autophagy	Pyroptosis	Ferroptosis	Cuproptosis
Biochemical characteristics		The caspase-mediated activation of oligonucleosomal DNA fragmentation	The ATP level was observed to decrease, while the activation of RIP1, RIP3, and MLK was noted	LC3 lipidation, formation of autophagosomes, increased autophagic flux, and Enhanced lysosomal activity were observed	Dependent or independent activation of Caspase-1, cleavage of GSDMD, and subsequent release of inflammatory factors	The process involves the reduction of GSH inhibition of GPX4, iron accumulation and lipid peroxidation.	Abnormal oligomerization of DLAT and inhibition of the level of Fe-S cluster proteins
Morphological characteristics		Cell shrinkage, preservation of membrane integrity, nuclear condensation and DNA fragmentation within the nucleus	Organelles exhibit swelling, cell membranes undergo rupture, and both cytoplasm and nuclei display disintegration	The cell membrane structure remains intact, while the cytoplasm exhibits vacuolation	Cell membrane disruption, cellular swelling and expansion with vesicle-like protrusions, preservation of nuclear integrity, and DNA fragmentation	The mitochondria become smaller, the density of the cell membrane increases, and the cristae of the mitochondria decrease	Mitochondrial contraction, cell membrane rupture Endoplasmic reticulum damage, and chromatin rupture
Core regulatory genes	Positive	Bax, Bak, Caspase, P53	RIPK1, RIPK3, MLKL	LC3, Beclin-1, ATG5, ATG7	Caspase-1, Capase-11, GSDMD	TFR1, ACSL4, P53	FDX1, LIAS, DLAT
	Negatie	Bcl-2, Bcl-xL	Caspase-8, FADD, A20	mTOR, AKT	GPX4	GPS4, SLC7A11, FSP1-CoQ10	SLC31Al, CTR1, ATP7, ATP8
Immune characteristics		Anti-Inflammatory	Pro-Inflammatory	Anti-Inflammatory	Pro-Inflammatory	Pro-Inflammatory	Pro-Inflammatory

## 3 Pathological association and interaction mechanism between different forms of PCD and AD

A core pathological feature of AD includes the cleavage of amyloid precursor protein (APP) by β-secretase and γ-secretase, resulting in the formation of neurotoxic β-amyloid oligomers (Aβ oligomers). Aβ is A polypeptide containing 39 to 43 amino acids, which is produced by the proteolytic action of APP by β-secretase and γ-secretase (Jucker and Walker, 2023). Aβ can be divided into monomers, oligomers, fibrils and amyloid fibers. Studies have shown that Aβ monomer itself does not directly affect neuronal function, but its hydrolysis and formation of soluble oligomers is the key factor in the development of AD (Jucker and Walker, 2023). In addition, Tau protein is a microtubule-associated protein encoded by a single gene, which is mainly distributed in hippocampal neurons. Its main physiological function is to promote the polymerization of microtubule-associated proteins in neurons and maintain the stability of microtubule structure, thereby ensuring the realization of normal physiological functions of neurons. Under normal physiological conditions, Tau protein has fewer phosphorylation sites. However, under pathological conditions, Tau protein is hyperphosphorylated and aggregated to form neurofibrillary tangles (NFTs), which in turn blocks nerve signal transmission and neurotransmitter transport, leading to the occurrence of AD (Mueller et al., 2021). The cascade reaction caused by Aβ deposition and Tau protein hyperphosphorylation promotes the formation of neurofibrillary tangles (NFTS) in the brain, which further aggravates the development of AD. It is noteworthy that different types of PCD pathways can participate in and aggravate this cascade reaction.

### 3.1 The mechanism of apoptosis and AD

Aβ can bind to Fas and TNF-α receptors on the cell membrane of neurons and activate the endogenous apoptosis signaling pathway. For example, after binding to Fas receptor, Aβ induces the expression and release of Fas ligand (FasL), forming Fas-fasl complex and activating caspase-8 to initiate apoptosis. Studies have shown that in AD model mice, the expression of Fas receptor in hippocampal neurons is upregulated, which is positively correlated with the degree of Aβ deposition. Meanwhile, administration of FasL antagonist can reduce neuronal apoptosis and improve cognitive function. In addition, Aβ deposition directly acts on mitochondria, increasing membrane permeability and releasing pro-apoptotic factors such as Cytc. Cytc binds to Apaf-1 and activates caspase-9 to initiate the mitochondria-mediated apoptotic pathway (Kumari et al., 2023). At the same time, Aβ deposition can also induce the production of A large number of ROS, which triggers oxidative stress. It attacks DNA, proteins and lipids, causing oxidative damage. It further activates the apoptotic signaling pathway in the cell, which eventually leads to apoptosis. Tau binds to Thr212/Thr231/Ser262 and other sites, which will dissociate from microtubules and lead to hyperphosphorylation. With the release of Cytc, Tau binds to Apaf-1 to form apoptotic bodies and activate the cascade of Caspase-9/3 (Alonso et al., 2010). In addition, phosphorylated Tau protein decreased the activity of PI3K/Akt pathway, increased the expression of pro-apoptotic factor Bax, and decreased the expression of anti-apoptotic factor Bcl-2 by competitively inhibiting the regulation of β-catenin by GSK-3β (Ulamek-Koziol et al., 2020). Notably, Caspase-3 can cleave Asp421 site of Tau protein to generate truncated Tau protein, which further aggravates the apoptotic process (Alonso et al., 2010).

### 3.2 Mechanisms of necroptosis and AD

In neurodegenerative diseases, necroptosis is mediated by A series of signaling pathways. Firstly, necroptosis can lead to the loss and damage of neurons, which further aggravates the deposition of Aβ and the formation of neurofibrillary tangles. Secondly, necroptosis can release inflammatory factors, such as IL-1β and TNF-α, activate microglia and astrocytes, trigger neuroinflammatory responses, and further damage the function and survival of neurons. Among them, the RIPK1/RIPK3/MLKL signaling axis is the key regulatory pathway. In the brain of AD patients, Aβ binds to the receptor on the cell membrane, activates the downstream RIPK1, and then phosphorylates RIPK3, leading to the phosphorylation and oligomerization of MLKL. Finally, Aβ inserts into the cell membrane to form holes, causing the release of cell contents and inducing the occurrence of cell necroptosis. In addition, cell necroptosis can also affect the intracellular REDOX balance, increase the production of ROS, and lead to oxidative stress damage, thereby promoting the aggregation of Aβ and neurotoxicity. Other studies have shown that p-MLKL, a necrotizing marker of apoptosis, co-localizes with Tau pathology in AD models. Inhibition of RIPK1 or MLKL can significantly reduce Tau aggregation and improve cognitive function (Balusu and De Strooper, 2024).

### 3.3 Mechanisms of pyroptosis and AD

Aβ and neuronal pyroptosis is A two-way regulation mechanism, which depends on NLRP family proteins in cultured neurons in cortical regions. When NLRP3 inflammasome activity is inhibited, Aβ deposition is significantly reduced. Dempsey et al. found that the use of NLRP3 inhibitor MCC950 can effectively inhibit the expression of NLRP3 and its downstream Caspase-1, thereby inhibiting pyroptosis and reducing Aβ accumulation (Dempsey et al., 2017). In addition to inhibiting NLRP3, pyroptosis can also be inhibited by inhibiting other inflammasomes such as NLRP1, NLRC4 and AIM2, which in turn reduces Aβ deposition (Tan et al., 2014). During the progression of AD, pyroptosis of microglia promotes the formation of ASC-Aβ complexes, not only exacerbating the accumulation of Aβ but also leading to the assembly of NLRP3 inflammasomes, activation of caspase-1, maturation of IL-1β, and cleavage of GSDMD, and further promoting pyroptosis of adjacent microglia. Tau protein showed similar to amyloid Aβ activation mechanism of NLRP3 inflammatory corpuscle. Specifically, phagocytosis of Tau by microglia disrupts lysosomal stability, leading to the release of cathepsin B and its binding to the NLRP3 inflammasome, thereby participating in its activation. This process induces the secretion of interleukin-1β (IL-1β) in an ASC-dependent manner (Stancu et al., 2019). When NLRP3 or ASC is deficient, Tau phosphorylation is significantly inhibited, and key kinases that induce Tau phosphorylation, such as CaMKII-α and GSK-3β, are significantly downregulated, while PP2A, a phosphatase that promotes Tau dephosphorylation, is significantly upregulated. This suggests that NLRP3 inflammasome can promote the pathological process of Tau by regulating the phosphorylation level of tau protein (Wang et al., 2020).

### 3.4 Mechanisms of ferroptosis and AD

In the ferroptosis pathway, the 5′untranslated region of APP mrna contains an Iron Response Element (IRE) that is regulated by intracellular iron levels. The expression of APP can be regulated by post-transcriptional Iron Regulatory Protein (IRP). When intracellular iron overload occurs, iron ions bind to IRP and promote its dissociation from IRE, thereby increasing the translation efficiency of APP, leading to the increase of APP protein level and finally the production of excess Aβ (Belaidi et al., 2018). As an important proprotein convertase, Furin protease can convert a variety of precursor proteins into mature proteins. The presence of iron ions can reduce the transcription and translation level of Furin, inhibit the activity of α-secretase and activate β-secretase, thus playing a key role in the processing of APP (Guillemot et al., 2013). Studies have shown that Aβ can promote ferroptosis by activating signaling pathways such as mGLIUS and STIM proteins, and regulating the expression of ferroptosis-related genes such as FTH1 and SAT1. The interaction between ferroptosis and Tau protein also shows the characteristics of bidirectional regulation. The imbalance of iron metabolism activates glycogen synthase kinase-3β (GSK-3β), promotes abnormal phosphorylation of Tau protein, inhibits the ubiquitin-proteasome system, and hinders the degradation of Tau protein, thereby accelerating its pathological aggregation (Wang et al., 2022a). In addition, ferroptosis-inducing agents further aggravate the toxic effects of Tau by inhibiting System Xc- and GPX4 (Wang et al., 2022b).

### 3.5 Mechanism of cuproptosis and AD

Cuproptosis is a novel form of cell death, which is associated with the abnormal accumulation of intracellular copper ions. Studies have shown that copper content is significantly increasedin peripheral blood and brain tissue of AD patients (Zhou et al., 2022). Excessive copper ions can bind to lipoic acid esterified proteins in mitochondria, leading to mitochondrial dysfunction and the loss of iron-sulfur cluster proteins, which in turn induces proteotoxic stress and further promotes the deposition and aggregation of Aβ. Aβ deposition is not only one of the pathological features of AD, but also may affect the occurrence of cuproptosis through A variety of mechanisms. On the one hand, Aβ deposition can lead to increased levels of oxidative stress in cells, and oxidative stress is one of the important factors inducing the death of copper. On the other hand, Aβ deposition may interfere with the distribution and metabolism of intracellular copper, increase the accumulation of copper ions in cells, and thus promote the occurrence of cuproptosis. Studies have shown that Aβ peptide and APP bind to copper through specific amino acid residues (such as histidine His13, His14, His6 and tyrosine Tyr10), and reduce Cu^2+^ to Cu^+^ through CTR1. This process generates ROS, which in turn leads to oxidative damage of Aβ peptide, intracellular proteins and lipids. And aggravate neurotoxicity (Multha et al., 1996). Knockout of CTR1 can significantly reduce the accumulation of copper in the brain, thereby alleviating Aβ42-induced neurodegeneration and enhancing the resistance to oxidative damage. Inhibition of CTR1 can effectively regulate the accumulation of copper in the brain and improve Aβ-induced neurotoxicity (Lang et al., 2013).

### 3.6 Mechanisms of autophagy and AD

In recent years, autophagy has become A hot spot in AD research. In AD, autophagy participates in the clearance of Aβ and promotes the degradation of p-tau, which is of greatsignificance for the improvement of AD (Weller and Budson, 2018). Specifically, the autophagy-lysosome pathway plays an important role in the metabolism of Aβ, and intracellular Aβ is mainly degraded by lysosomal metabolism. Under normal circumstances, Aβ produced by autophagosomes is transported to lysosomes and degraded by A variety of hydrolases. Therefore, the amount of Aβ in neuronal lysosomes is very small. However, under pathological conditions, the content of Aβ in lysosomes increases significantly due to the blockage of this pathway, and the excessive deposition of Aβ and APP will lead to autophagy dysfunction, and the clearance of Aβ by autophagy will fall into A decompensated stage. With the continuous exploration of the pathogenesis of AD, it has been found that inducing autophagy in the early stage of AD can switch a large number of APP-rich metabolic substrates to the autophagy-lysosome system metabolic pathway, accelerate the clearance of misfolded proteins and damaged organelles, and thus improve AD (Whyte et al., 2017). Autophagy is also one of the key mechanisms to clear phosphorylated tau protein from neurons. Studies have shown that the damage of autophagy-lysosome system can lead to the formation of tau oligomers and insoluble aggregates. Autophagy dysfunction is closely related to the excessive accumulation of p-tau in the brain, which can be significantly alleviated by inducing autophagy (Zhang J. et al., 2024; Silva et al., 2019). Studies have found that activation of AMPK-mTOR pathway can enhance the autophagy pathway to reduce the content of tau protein, thereby improving the cognitive impairmentof AD mice (Malampati et al., 2020). In addition, some studies have suggested that activating the Nrf2/Keap1/NQO-1 pathway can increase autophagy flux and promote the clearance of P-tau, thus playing a therapeutic rolein AD (Ma et al., 2024).

## 4 Mechanisms of interaction between different PCDS

Multiple modes of PCD and their combination play important roles in the development and progression of AD. As a key node in the turnover of old and new cells, autophagy plays a central role in the regulation of AD by crosstalk with a variety of PCD pathways. Autophagy is a form of PCD that involves the encapsulation of intracellular proteins or organelles into autophagic vesicles, which fuse with lysosomes to achieve the degradation of their contents and the recycling of autophagic substrates, and ultimately trigger cell self-digestion (Fleming et al., 2022). In addition, autophagy can interact with various forms of PCD such as apoptosis, necroptosis, pyroptosis, ferroptosis, and cuproptosis in AD.

### 4.1 Autophagy-apoptosis-necroptosis

Autophagy has been confirmed as an important physiological protective mechanism in cells. In the early stage of disease, the body actively activates autophagy pathway to prevent cell apoptosis. However, with the progression of the disease, when autophagy function is impaired, it may lead to a significant increase in neuronal apoptosis. In recent years, more and more studies have shown that the excitability and toxicity of glutamate play a key role in neurodegenerative diseases. It has been reported that the activation of autophagy is closely related to the level of glutamate. For example, in a cell model of glutamate-induced AD, glutamate stimulation significantly upregulated the expression of autophagy-related proteins Beclin-1 and LC3-II, decreased the Bax/Bcl-2 ratio and P62 protein level, and restored mitochondrial membrane potential. These results indicate that the increased level of autophagy can effectively inhibit the expression of apoptosis-related proteins and reduce the deposition of β-amyloid (Aβ), Tau protein phosphorylation and the formation of neurofibrillary tangles (NFTs), which provides A new theoretical basis (Xie D. et al., 2023). In addition, IKKβ-activating enzyme has been shown to inhibit the occurrence of necroptosis by enhancing autophagy activity in Aβ-induced neuroblastoma cell model. Specifically, when autophagy-related proteins were silenced, the expression of necrotizing effectors in cells was significantly increased, further exacerbating the necrotizing process. These results indicated that autophagy could effectively inhibit RIPK-mediated necroptosis in Aβ-induced neuroblastoma cells. Further studies have found that IKKβ is an upstream signaling molecule of autophagy, and its activation can significantly improve necroptosis caused by impaired autophagy and reduce Aβ deposition, which has potential value for the treatment of AD (Wang W. et al., 2022).

### 4.2 Autophagy-necroptosis-ferroptosis

Previous studies have found that necroptosis in rats with Aβ-induced AD can increase the expression of SLC7A11, the key factor of ferroptosis, and decrease the level of TFR, leading to the decrease of RIPK1 and RIPK3 expression, and thus improve the pathological process of AD mediated by necroptosis. Further studies have found that necrospot-1, an inhibitor of necroptosis, has no effect on ferroptosis, but Aβ induced necroptosis is inhibited by ferrostatin, indicating that ferroptosis pathway may act as an upstream signal of necroptosis to regulate Aβ neurotoxicity in AD (Tang et al., 2021).

### 4.3 Autophagy-ferroptosis - cuproptosis

**FIGURE 3:**
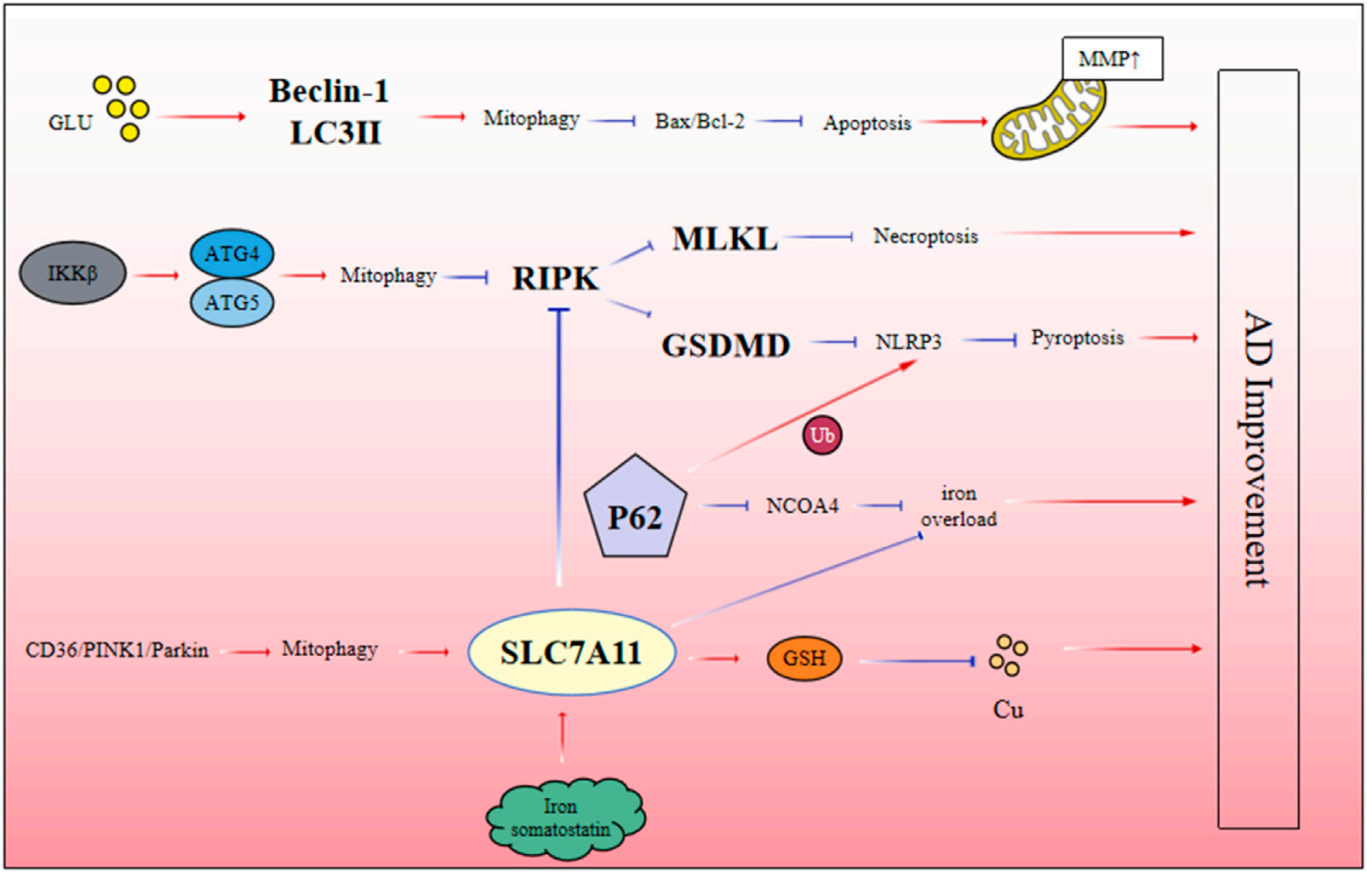
The interaction mechanism between different forms of PCD.

Autophagy plays a key role in the regulation of ferroptosis. Excess iron interacts with ferritin and binds to NCOA4 to induce selective autophagy and release more free iron. At the same time, P62 can lead to abnormal expression of ferritin autophagy, resulting in large iron accumulation and iron overload (Quiles and Mancias, 2019). At the same time, there is crosstalk between mitophagy and ferroptosis in the process of AD. By establishing A cell model of Aβ-induced AD damage, we found that there was a crosstalk between CD36/PINK/Parkin-mediated mitophagy and ferroptosis. This ferroptosis pathway could be exacerbated by mitophagy inhibitors. These results indicate that CD36/PINK/Parkin-mediated mitophagy plays an important role in regulating ferroptosis and inhibiting the deposition of Aβ in AD (Li et al., 2022). Therefore, dysregulation of the autophagy system may directly lead to the abnormal accumulation of iron in the brain of AD, which in turn triggers ferroptosis. Notably, SLC7A11, a key inhibitor of ferroptosis, is also closely related to cuproptosis. Studies have found that when the expression level of SLC7A11 is upregulated, it can promote the synthesis of glutathione from cysteine, which in turn chelates copper ions, reduce the level of intracellular copper ions, and inhibit the occurrence of cuproptosis (Zhang P. et al., 2024). Although there are few reports on the crosstalk relationship among autophagy, ferroptosis and cuproptosis in AD, using SLC7A11 as a cross point to link autophagy, ferroptosis and cuproptosis can provide clues for future research on the pathogenesis of AD (Figure 3).

## 5 Plant metabolites of traditional Chinese medicine regulate the pathways of PCD in AD cells

**FIGURE 4:**
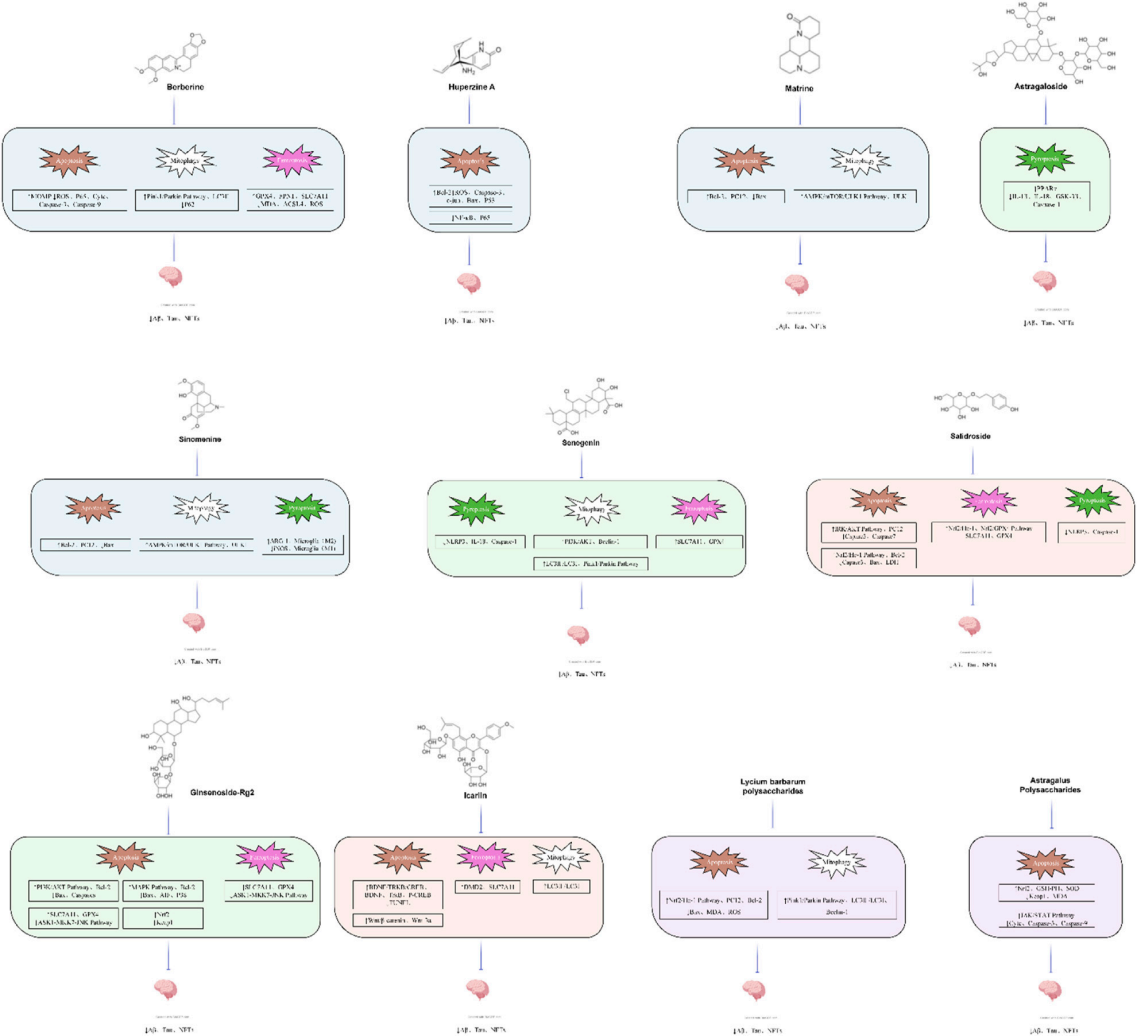
The chemical structure of natural active components.

This review summarizes recent advances in PCD in AD. Subsequently, we summarized and analyzed the experimental studies, recent reviews and network pharmacological analysis literature on the use of plant metabolites of traditional Chinese medicine in the treatment of AD in the past decade. In this review, we summarized the efficacy and mechanisms of plant metabolites of traditional Chinese medicine in regulating PCD (Figure 4) (Tables 2–6).

**TABLE 2:** Regulation of apoptosis by active components of traditional Chinese medicine.

Target of action	Experimental subjects	Possible mechanisms	Natural plant molecules	References
Apoptosis	HT22 cells	↑MOMP ↓ROS, P65, Cytc, Caspase-3, Caspase-9	Berberine	Zhang et al. (2024c)
	N2a cells	↑Bcl-2 ↓ROS, Caspase-3, c-jun, Bax, P53	Huperzine A	Wang et al. (2023)
	Aβ-included astrocyte	↓NF-κB, P65	Huperzine A	Tao et al. (2013)
	APP/PS1 mice	↑Bcl-2, PC12, ↓Bax	Matrine	Yan et al. (2007)
	Lipopolysaccharide -induced macrophages	↓Bax, Caspase-3	Sinomenine	Lu et al. (2023)
	PC-12 cells	↑Nrf2/Ho-1 Pathway, PC12, Bcl-2 ↓Bax, MDA, ROS	Lycium barbarum polysaccharides	Bao et al. (2024)
	APP/PS1 mice	↑Nrf2, GSH-PH, SOD ↓Keap1, MDA	Astragalus polysaccharides	Zhao et al. (2024)
	*Drosophila melanogaster*	↑JAK/STAT Pathway ↓Cytc, Caspase-3, Caspase-9	Astragalus polysaccharides	Qin et al. (2020)
	ICR mice	↑Bcl-2 ↓Bax, Caspase-3, Caspase-9	Ginsenoside	(Wang et al., 2021), (Luong et al., 2021)
	HT22 cells	↑MAPK Pathway, Bcl-2 ↓Bax, AIF, P38	Ginsenoside	Hou et al. (2017)
	Aβ-included mice	↑PI3K/AKT Pathway, Bcl-2 ↓Bax, Caspases	Ginsenoside	Fernandez-Moriano et al. (2017)
	hydrobromide-Induced mice	↑Nrf2 ↓Keap1	Ginsenoside	Kim et al. (2019)
	Aβ-included mice	↑BDNF/TRKB/CREB, BDNF, TrkB, P-CREB ↓TUNEL	Icarin	Tao et al. (2021)
	APP/PS1 mice	↑Wnt/β-catenin, Wnt-3a	Icarin	Zeng et al. (2022)
	PC-12 cells	↑ERK/AKT Pathway, PC12 ↓Capase3, Caspase7	Salidroside	Hu et al. (2024a)
	Aβ-included mice	↑Nrf2/Ho-1 Pathway, Bcl-2 ↓Capase3, Bax, LDH	Salidroside	Liao et al. (2019)

**TABLE 3:** Regulation of Pyroptosis by active components of traditional Chinese medicine.

Target of action	Experimental subjects	Possible mechanisms	Natural plant molecules	References
Pyroptosis	Lipopolysaccharide -induced macrophages	↑ARG-1, Microglia (M2) ↓iNOS, Microglia (M1)	Sinomenine	Lu et al. (2023)
	AβO-included mice	↑PPAR gamma ↓IL-1β, IL-18, GSK-3β, Caspase-1	Astragaloside	Shen et al. (2023)
	MPTP-included mice	↓NLRP3, IL-1β, Caspase-1	Senegenin	Tian et al. (2022)
	Aβ-included mice D-galactose-included Mice and PC12 cells	↓NLRP3, Caspase-1	Salidroside	Xie et al. (2020)

**TABLE 4:** Regulation of Autophagy by active components of traditional Chinese medicine.

Target of action	Experimental subjects	Possible mechanisms	Natural plant molecules	References
Autophagy	APP/PS1 mice	↑Pink1/Parkin Pathway, LC3Ⅱ ↓P62	Berberine	Zhang et al. (2023a)
	Neonatal mice	↑AMPK/mTOR/ULK1 Pathway, ULK1	Matrine	Xie et al. (2016)
	MPTP-included mice	↑Beclin-1, LC3Ⅱ/LC3Ⅰ ↓PI3K/AKT/mTOR Pathway, P62	Sinomenine	Hou et al. (2024)
	LPS-induced mice	↑Pink1/Parkin Pathway, LC3Ⅱ/LC3Ⅰ, Beclin-1	Lycium barbarum polysaccharides	Toldo and Abbate (2024)
	PC12 cells	↑PI3K/AKT, Beclin-1	Senegenin	(Yan et al., 2024), (Zhang et al., 2023b)
	HT22 cells	↑LC3Ⅱ/LC3Ⅰ, Pink1/Parkin Pathway	Senegenin	Bieri et al. (2018)
	D-galactose-included PC12 cells	↓ PC12 ↑LC3Ⅱ/LC3Ⅰ	Icarin	Xiao et al. (2022)

**TABLE 5:** Regulation of Ferroptosis by active components of traditional Chinese medicine.

Target of action	Experimental subjects	Possible mechanisms	Natural plant molecules	References
Ferroptosis	3 × Tg-AD mice	↑GPX4, FPN1, SLC7A11 ↓MDA, ACSL4, ROS	Berberine	Wang et al. (2022c)
	D-gal-induced PC-12 cells	↑SLC7A11, GPX4 ↓ASK1-MKK7-JNK Pathway	Ginsenoside	Cui et al. (2021)
	PC-12	↑ACSL4, GPX4	Senegenin	Li et al. (2023c)
	Aβ-included mice	↑Nrf2/Ho-1, Nrf2/GPX4 Pathway, SLC7A11, GPX4	Salidroside	(Cai et al., 2021), (Shuangmei, 2021)
	APP/PS1 mice	↑DMD2, SLC7A11	Icarin	Liu et al. (2018)

**TABLE 6:** Regulation of cuproptosis by active components of traditional Chinese medicine.

Target of action	Experimental subjects	Possible mechanisms	Natural plant molecules	References
Cuproptosis	APP/PS1 mice	↓P-tau,ROS,GSK-3β	Curcumin	Li et al. (2025)
	HT22 cells	↓P-tau,ROS	Quercetin	Yang et al. (2013)
	Tg mice	↓P-tau,ROS	Epigallocatechin gallate	Shen et al. (2018)

### 5.1 Alkaloids (primarily regulate apoptosis and autophagy via “Nrf2/AMPK/mTOR”)

#### 5.1.1 Berberine

Berberine is a plant metabolites commonly found in rhizome of *Coptis chinensis Franch [*Ranunculaceae*]* and *Scutellaria baicalensis Georgi [*Lamiaceae*]*, Berberine’s isoquinoline structure enables it to penetrate cell membranes, interact with multiple targets, and exert antioxidant and anti-inflammatory effects. It can also have beneficial effects on neurovascular degeneration by protecting blood vessels. Numerous studies have shown that berberine can reduce Aβ deposition and the formation of NFTs in A phosphorylated way of Tau protein. It has the ability to cross the blood-brain barrier, reduce inflammation, reduce oxidative damage, protect neurons, and reduce neurotoxicity in the brain. A recent study reported that berberine not only improved cognitive impairment in AD mice, reduced neuronal damage, Aβ deposition, and Tau hyperphosphorylation, but also increased the expression levels of SOD, GSH, GPX4, FPN1, and SLC7A11, and reduced MDA, ACSL4, and ROS levels by regulating Nrf2 transcription levels. It can effectively reduce the abnormal accumulation of iron in the brain of mice, and finally reduce the deposition of Aβ (Li et al., 2023a). A recent study involving the protective effects of β-amyloid-induced neurotoxicity in HT22 cells found that berberine could inhibit apoptosis and intracellular ROS levels, increase mitochondrial membrane potential, and reduce the rates of p-p65/p65, Cytc and Caspase-9/3 cleavage, This may be related to the presence of an electrophilic nitrogen-containing heterocycle and aromatic ring in berberine, which allows it to bind with key intracellular proteins, regulate the cell cycle, and promote cell survival. Thus, berberine inhibited the mitochondrial pathway of cell apoptosis and effectively reduced Aβ deposition (Zhang R. L. et al., 2023). In addition, berberine was found to improve cognitive function in AD mice by inducing autophagy and reducing the pathological accumulation of Aβ and Tau through activating the PINK/Parkin signaling pathway. At the same time, berberine has strong lipophilicity, which can promote the removal of damaged mitochondria and reduce oxidative stress and inflammation, thereby protecting neurons from injury. This finding provides an important theoretical basis for the application of berberine in the treatment of AD (Wang et al., 2023).

#### 5.1.2 Huperzine A

Huperzine A is a highly effective, specific and reversible cholinesterase inhibitor, which is extracted and isolated from *Huperzia serrata (Thunb.) Trevis [*Lycopodiaceae*]*. Due to the presence of a pyridine ring in its structure, it is able to form hydrogen bonds with the acidic amino acids in the active site of acetylcholinesterase, enabling it to bind tightly to the enzyme’s active site. This interaction inhibits the hydrolysis of acetylcholine, thereby slowing its degradation in the synaptic cleft. As a result, acetylcholine’s activation of its receptors is enhanced, improving the efficiency of neurotransmission and ultimately contributing to the improvement of cognitive function (Friedli and Inestrosa, 2021). Huperzine A has been shown to attenuate neuronal apoptosis by reducing ROS generation and Caspase-3 activity in Aβ25-35 and crissorine induced apoptosis models. This may be due to the strong electron-donating effect of the aromatic ring in its molecule, which can react with free radicals through hydrogen atom donation, thereby reducing the damage caused by free radicals to cells. Huperzine A, due to its aromatic ring structure and nitrogen-containing heterocycle, interacts with specific receptors on the cell membrane, regulating intracellular signaling pathways. It upregulates the anti-apoptotic protein Bcl-2 and downregulates the expression levels of pro-apoptotic factors c-jun, Bax, and P53, further reducing the occurrence of cell apoptosis (Tao et al., 2013). Other studies have shown that huperzine A may promote the transformation of APP to non-amyloid pathway by activating MAPK signaling pathway, increase the production of soluble APP, and reduce the accumulation of β-amyloid peptide (Aβ), thus playing a neuroprotective role (Yan et al., 2007). In addition, huperzine A can also prevent D-galactose induced neurovascular injury and blood-brain barrier dysfunction by preventing nuclear translocation of NF-κB, thus effectively preventing neuronal apoptosis (Xie et al., 2016). These findings demonstrate the extensive neuroprotective effects of huperzine A across various forms of PCD. This neuroprotection is achieved through its molecular structure, which enables it to interact with different cellular targets and signaling pathways, providing a solid theoretical foundation for its application in AD treatment.

#### 5.1.3 Matrine

Matrine is an alkaloid extracted from *Sophora flavescens Aiton [*Fabaceae*]*, which has a variety of pharmacological effects such as anti-apoptosis, anti-oxidation and immune regulation, and is widely used in the treatment of neurodegenerative and cardiovascular diseases. Previous studies have shown that oxidative stress and apoptosis are two key factors in the pathogenesis of AD, and they are both involved in its pathological process. Specifically, the formation of Aβ and senile plaques can activate oxygen free radicals, leading to A decrease in the activity of antioxidant enzymes such as superoxide dismutase (SOD) and glutathione peroxidase (GSH-Px), thereby reducing the ability to remove malondialdehyde (MDA) and free radicals, initiating neuronal apoptosis, and inhibiting the expression of the anti-apoptotic factor Bcl-2. At the same time, it activates the expression of pro-apoptotic factor Bax, which eventually leads to neuronal dysfunction. The *in vitro* model of PC12 cells induced by Aβ25-35 showed that the viability of PC12 cells was significantly decreased, and the levels of oxidative stress and apoptosis were increased, suggesting that the occurrence of AD may be closely related to oxidative stress and apoptosis caused by Aβ deposition. Further studies have shown that the indole ring and nitrogen-containing heterocycle in matrine confer significant lipophilicity, enabling it to traverse cell membranes and interact with specific intracellular receptors and enzymes. The electron-donating properties of the aromatic ring and nitrogen heterocycle allow matrine to donate hydrogen atoms, thereby effectively scavenging peroxides and free radicals within the body. This could effectively inhibit oxidative stress and apoptosis, thereby reducing the damage to PC12 cells induced by Aβ25-35, suggesting its potential value in the treatment of AD (Zhang et al., 2020). In addition, matrine can enhance mitophagy by activating AMPK/mTOR/ULK1 signaling pathway, thereby reducing neuronal apoptosis and inflammatory response and improving nerve injuryin rats (Li Aaqi, 2024). In addition, matrine can inhibit pyroptosis and reduce the release (Lu et al., 2023) of inflammatory factors in the central nervous system by inhibiting the expression of GSDMD protein.

#### 5.1.4 Sinomenine

Sinomenine is an isoquinoline alkaloid extracted from the roots and stems of *Sinomenium acutum (Thunb.) Rehder and E.H.Wilson [*Menispermaceae*]*, and its derivatives have been extensively studied as bioactive agents. Existing studies have shown that sinomenine exerts broad pharmacological effects, including anti-tumor, anti-inflammatory, neuroprotective, and immunosuppressive properties, through the aromatic and nitrogen-containing heterocyclic structures in its molecule. These effects are mediated by its influence on signaling pathways such as PI3K/Akt/mTOR, NF-κB, MAPK, and JAK/STAT. For this reason, sinomenine also plays an important role in cardio-cerebrovascular protection and organ protection through NF-κB, Nrf2, MAPK and PI3K/Akt/mTOR signaling pathways (Hou et al., 2024). Specifically, sinomenine could inhibit the expression of iNOS, increase the level of ARG-1, and promote the polarization of macrophages from M1 to M2, thereby reducing neuroinflammation. At the same time, sinomenine could increase the expression of type II collagen and aggrecan, reduce the level of ROS and MDA, increase the activity of SOD, enhance the antioxidant capacity of the body, and inhibit cell apoptosisby down-regulating the expression of Bax, Caspase-3, MMP-2/9 and iNOS (Hou et al., 2022). In addition, sinomenine can inhibit the PI3K/Akt/mTOR pathway, upregulate the ratio of Beclin1 and LC3-II/LC3-I and downregulate the expression of P62, promote the autophagy of dopaminergic neurons to improve the activity of neurons, and exert its potential pharmacological effects (Bao et al., 2024).

### 5.2 Polysaccharides (primarily regulate apoptosis, autophagy and ferroptosis via “Nrf2/Pink1/GPX4”)

#### 5.2.1 Lycium barbarum polysaccharide

Lycium barbarum polysaccharide is a water-soluble polysaccharide extracted from *Lycium chinense Mill [*Solanaceae*]*,which is considered to be the commonly metabolite in *L. chinense Mill [*Solanaceae*]*. Lycium barbarum polysaccharide are composed of various monosaccharide units, such as glucose, mannose, arabinose, and galactose, which are linked by specific glycosidic bonds to form polysaccharide chains with a defined spatial conformation. This intricate structure endows the polysaccharides with a range of biological activities, including antioxidant, anti-apoptotic, autophagy-inducing, and neuroprotective effects. Specifically, Lycium barbarum polysaccharide contain multiple hydrogen bond donors and electron-donating groups that can interact with free radicals, thereby exhibiting strong antioxidant activity. This enables them to effectively scavenge free radicals and concurrently inhibit various forms of PCD. Previous studies have shown that LBP can effectively inhibit oxidative stress in the hippocampus and significantly improve scopolamine-induced cognitive and memory impairment by reducing the ratio of Bax/Bcl-2 (Zhao et al., 2017). Current studies have shown that LBP exhibits protective effects against neurotoxicity by up-regulating Nrf2/HO-1 signaling, and the enhancement of Nrf2/HO-1 signaling helps to inhibit HO_2_-induced_2_ PC12 cell damage while ameliorating oxidative stress and ameliorating apoptosis (Cao et al., 2017). In addition, LBP also showed a significant neuroprotective effect on glutamate-induced apoptosis. Further studies showed that LBP played a protective role against neurotoxicity by up-regulating the Nrf2/HO-1 signaling pathway, which helped to improve oxidative stress and cell apoptosis (Jiang et al., 2023). By (Zhenghao et al., 2024) activating SLC7A11/GPX4 signaling pathway, LBP also enhanced GPX4 activity, reduced ferroptosis induced by lipid peroxidation, and reduced Aβ deposition. In the brain tissue of AD patients, the disorder of mitophagy leads to mitochondrial morphology, structure and function abnormalities, which in turn leads to cell damage and neuronal death (Zhao et al., 2024). Furtherstudies have shown that with the disorder of autophagy function in vivo, excessive β-amyloid (Aβ) protein is observed in the brain of AD patients, suggesting that the disorder of mitophagy may be closely related to the pathogenesis and progression of AD (Um et al., 2024). In a study on the effect of β1-42 on the activity of SH-SY5Y cells, it was found that the protein expressions of Pink1, Parkin, LC3-II/LC3-I and Beclin-1 were significantly increased, while P62 protein expression was decreased after intervention with LBP. These results suggest that LBP may reduce the damage of SH-SY5Y cells induced by Aβ 1–42 and maintain the metabolic balance by regulating mitophagy through its unique sugar chain structure, which has potential important significance for the prevention of AD (Qin et al., 2025).

#### 5.2.2 Astragalus polysaccharide

Astragalus membranaceus is the dried root of the leguminous plant *Astragalus mongholicus Bunge [*Fabaceae*]*,which has the functions of tonifying qi, rising Yang, solidifying surface, antiperspirating, and reducing swelling. Astragalus polysaccharide a representative metabolite present in *stragalus mongholicus Bunge [*Fabaceae*]*. As one of the common metabolites, Astragalus polysaccharide has shown a variety of biological effects in the central nervous system, such as anti-inflammatory, anti-oxidation, immunomodulation and neuroprotection. Studies have shown that astragalus polysaccharides can activate the Nrf2 signaling pathway through its unique sugar chain structure, promoting its translocation to the nucleus and inhibiting its expression in the cytoplasm. This process restoring the expression levels of Keap1, SOD, GSH-Px and MDA, and significantly reducing cell apoptosis and Aβ deposition. It can also significantly improve the cognitive ability of APP/PS1 mice (Qin et al., 2020). In a study on metabolic stress in AD, Astragalus polysaccharide not only significantly reversed the abnormal body weight and insulin level, but also improved the overall metabolic status. It also reduced the number of activated microglia and astrocytes around amyloid plaques, thereby reducing neuroinflammation. Notably, Astragalus polysaccharidedid not directly reduce Aβ deposition, but rather improved the behavioral performanceof AD mice by counteracting metabolic stress and ameliorating metabolic stress-induced neuroinflammation (Huang et al., 2017). In addition, aging is an important factor that increases the risk of neurodegenerative diseases such as AD. In an anti-aging experiment in *Drosophila*, it was found that the β-1,3-glucan in astragalus polysaccharides can bind to important Toll-like receptors in the immune system, which significantly alleviate intestinal homeostasis imbalance, improve sleep disorders, and protect neurons by rescuing aging-induced JAK/STAT, Toll and IMD pathways, downregulating the expression of Cyt C and Caspase-3/9, and reducing apoptosis. It can also delay the onset of AD phenotype in Aβ42-induced AD flies (Li et al., 2023b).

### 5.3 Saponins (primarily regulate apoptosis, pyroptosis and autophagy via “STAT/PI3K//Pink1”)

#### 5.3.1 Astragaloside

Saponins can promote the growth and development of human immune organs through a variety of signaling pathways, regulate the activity of immune cells, and increase the secretion of immune-related cytokines and specific antibodies. The potential pharmacological effects of saponins on the central nervous system have been a research hotspot (Shen et al., 2023). *Astragalus mongholicus Bunge [*Fabaceae*]* is a commonly used traditional Chinese medicine for the treatment of AD. Astragalosides, among the mainly metabolites of astragalus membranaceus, have various biological functions such as regulating immunity, anti-oxidation, anti-apoptosis and inhibiting neurotoxins. Astragaloside, include astragaloside Ⅰ, astragaloside Ⅱ, astragaloside Ⅲ and astragaloside Ⅳ, among these, Astragaloside IV demonstrates the most optimal biological activity due to the synergistic effect of its extended sugar chain structure and the triterpene aglycone portion (Dai et al., 2020). Although this metabolite is frequently studied in the context of TCM, it is also present in other medicinal plants beyond TCM use, highlighting its broader pharmacological significance. Studies have shown that astragaloside Ⅳ, as A selective natural PPARγ agonist, inhibits BACE1 activity by activating and upregulating the expression of PPARγ, thereby enhancing the phagocytosis of Aβ and reducing the release of cytokines, ultimately reducing the level of Aβ in APP/PS1 mouse model (Shen et al., 2023). In addition, Wang et al. found that astragaloside Ⅳ can effectively block Aβo-induced neuronal pyroptosis and has A high binding affinity with pyroptosis-related protein Caspase-1, further revealing the specific mechanism of astragaloside Ⅳ in pyroptosis and its potential application value in the prevention and treatment of AD (Wang et al., 2021).

#### 5.3.2 Ginsenoside

*Panax ginseng C.A.Mey [*Araliaceae*]*. belongs to the family Araliaceae and comprises a group of plants with significant medicinal properties. As a common tonic traditional Chinese medicine, ginseng has a variety of pharmacological effects such as anti-fatigue, anti-virus and anti-oxidation. Ginsenoside, the main metabolites in ginseng, has been shown to have significant neuroprotective effects in animal and cell model studies (Luong et al., 2021). Ginsenosides can be divided into three groups according to differences in their chemical skeletons (aglycones) and sugar chain attachment sites:protopanaxtriol type (PPT; They include ginsenosides Rg1, Re, Rg2, Rh1, and Rf), protoginsenosides (PPD; Including ginsenoside Rb1, Rb2, Rd, Rg3, and Rh2) and oleanolic acid derivative forms (e.g., ginsenoside Ro) (Hou et al., 2017). After deglycosylation, protopanaxadiol-type ginsenosides Rd and Rb1 exhibit increased lipophilicity, enabling them to directly insert into cellular and mitochondrial membranes, thereby altering membrane lipid fluidity and lipid raft architecture. These changes can further influence the localization and conformation of membrane-associated proteins, such as members of the Bcl-2 family, TLR4, and TNFR, ultimately facilitating the regulation of key signaling pathways involved in apoptosis, autophagy, and inflammation. Caspase family is involved in the pathological process of neuronal apoptosis in AD. Studies have shown that ginsenoside Rd, Rb1 and Rg1 can effectively alleviate neuronal apoptosisinduced by hydrogen peroxide and β-amyloid protein (Aβ) by down-regulating the protein levels of Caspase-3 and Caspase-9 and increasing the ratio of Bcl-2/Bax (Hou et al., 2017; Fernandez-Moriano et al., 2017). In addition, ginsenoside Rb2, with reduced total sugar residues and decreased molecular polarity, exhibits enhanced lipophilicity, which enables it to more readily interact with cellular and mitochondrial membranes. This structural property facilitates its modulation of intracellular signaling cascades. Specifically, Rb2 has been shown to alleviate glutamate-induced neurotoxicity by suppressing the activation of p38 and preventing the translocation of Apoptosis Inducing Factor (AIF) within the mitogen-activated protein kinase (MAPK) signaling pathway. Through these mechanisms, Rb2 not only reduces neuronal apoptosis but also preserves mitochondrial integrity, thereby highlighting the critical role of its structural features in conferring neuroprotective effects.In addition, ginsenoside Rb2 can upregulate the expression of anti-apoptotic gene Bcl-2, reduce the level of pro-apoptotic gene Bax, and inhibit the apoptosis of cells mediated by AIF, so as to play a neuroprotective role and achieve the effectof treating AD. (Kim et al., 2019) CUI et al. showed that ginsenoside Rg2 can activate the PI3K/Akt signaling pathway in the hippocampus of AD rats induced by Aβ25-35, significantly increase the ratio of Bcl-2 to Bax in the hippocampus, and reduce the expression level of Caspase, thereby effectively improving the learning and memory ability of AD rats (Cui et al., 2021). In addition to its intervention effect on apoptosis, more and more studies have pointed out that the accumulation of autophagosomes in hippocampus and cortical axons leads to the block of autophagic flow and the failure of autophagosomes to properly fuse with the lysosomes around the cell body in the early stages of AD. Therefore, enhancing autophagy is considered to be a promising therapeutic strategy to deal with the toxicity of misfolded proteins. The protopanaxadiol-type metabolite ginsenoside CK, characterized by its low number of sugar moieties and high lipophilicity, can readily embed into membrane structures and induce oxidative modification of Keap1, thereby releasing Nrf2. This activation of the Nrf2/Keap1 signaling pathway upregulates antioxidant enzymes, enhances the cellular capacity to eliminate reactive oxygen species (ROS), and confers antioxidant and cytoprotective effects, ultimately improving memory function in AD mouse models (Yang et al., 2019). In addition, ginsenoside CK can also inhibit the ASK1-MKK7-JNK signaling pathway, reduce the expression of ferroptosis marker Ptgs2, and upregulate key proteins such as SLC7A11 and GPX4 to reduce the accumulation of unstable iron in the brain, thereby inhibiting ferroptosis, which helps to prevent and improve the occurrence and development of AD (Yan et al., 2024).

#### 5.3.3 Senegenin

Senegenin is the common bioactive components of *Polygala tenuifolia Willd [*Polygalaceae*],*which belong to the structure of pentocyclic triterpene saponins. The chemical properties of most senugenin are unstable, and it is easy to hydrolyze into senugenin, senugenin, polygallic acid, polygalactosenoside and various sugar groups under external conditions (Zhang L. et al., 2023). Because of its significant anti-aging and antioxidant pharmacological effects, Senegenin play a key role in the prevention and treatment of AD and related mechanisms. Studies have shown that Senegenin may interfere with pyroptosis, autophagy and ferroptosis through a variety of mechanisms, thereby effectively preventing and treating AD. With the development of AD, Aβ1-42 activates the PI3K/Akt signaling pathway in neuronal cells, leading to A significant decrease in autophagy function and the expression of Beclin-1, affecting the formation of autophagosomes, and further aggravating neuronal cell damage. At the same time, insufficient autophagy flux in the brain further leads to mitochondrial damage and Aβ protein deposition, which ultimately impairs cognitive function (Ren et al., 2022; Bieri et al., 2018). As an important component of autophagosome, LC3-II is closely related to autophagic flux (Wan et al., 2023). Senegenin predominantly possesses an oleanane/oleanolic-type triterpenoid aglycone skeleton, characterized by a hydrophobic plane that facilitates interaction with membrane phospholipids, lipid rafts, and cholesterol-rich microdomains. Deglycosylation and deacylation increase lipophilicity and enhance blood–brain barrier permeability; upregulate the expression of PINK1 and Parkin in neurons, promote the localization of Parkin in mitochondria, activate autophagy, and ultimately reduce the secretion of Aβ (Tian et al., 2022). In addition, according to its structure senegenin can inhibit pyroptosis and delay the progression of AD. Studies have shown that senegenin can cross the blood-brain barrier and upregulate the expression of SHP-2, thereby inhibiting the activation of NLRP3 and reducing the release of IL-1β (Pellegrini et al., 2019). Fan.et al.further confirmed that senegenin significantly reduced the expression and activation of pyroptosis factors NLRP3, Pro-caspase-1, Caspase-1, Pro-IL-1β and IL-1β, effectively inhibited pyroptosis and improved the symptoms of AD (Fan et al., 2017). The study found that exist in the brain iron deposition in patients with AD and iron balance the typical pathological features (Hannn Q. et al., 2023). A number of studies have shown that senegenin as the principal metabolites of *P. tenuifolia Willd*, polygalasaponins possess multiple glycosidic chains and galloyl substituents that confer substantial antioxidant capacity. Upon hydrolysis, the resulting aglycones or secondary metabolites more readily penetrate the nucleus and mitochondria, where they modulate oxidative stress. Through these actions, senegenin effectively suppress ferroptotic signaling cascades in neurons and thereby exert neuroprotective effects against AD. Importantly, these activities are closely associated with their structural features, as the balance between glycosylation, acyl substitution and aglycone exposure determines lipophilicity and the magnitude of their antioxidant and anti-ferroptotic effects (LI C. et al., 2023; Zhao et al., 2023). Experimental studies have shown that senegenin can inhibit the ferroptosis pathway in PC12 cells, significantly upregulate the expression of ferroptosis related proteins SLC7A11 and GPX4 in the brain of AD mice, prevent the occurrence of ferroptosis, correct the abnormal iron metabolism, and play A neuroprotective role in AD mice (Zhang H. et al., 2022).

### 5.4 Flavonoid (primarily regulate apoptosis and ferroptosis via “Nrf2/Ho-1/GPX4”)

#### 5.4.1 Icariin

*Epimedium brevicornu Maxim [*Berberidaceae*]* is a perennial herb of Epimedium genus in Berberidaceae, which has the functions of toning liver and kidney, strengthening muscles and bones, eliminating wind and dampness, and invigorating essence and qi. Studies have shown that the extracts of *E. brevicornu Maxim* contain more than 260 metabolites, among which the main metabolites icariin has been shown to have significant antioxidant, anti-inflammatory and immunomodulatory effects (Tao et al., 2021). Recent studies have further revealed the potential of icariin in the treatment of central nervous system diseases, such as AD, Parkinson’s disease and ischemic stroke, and its mechanism involves multi-target and multi-pathway neuroprotective effects (Zeng et al., 2022). Specifically, icariin exhibits amphiphilic properties due to the balance between its methoxy group and glycosidic moieties, whereby the methoxy substitution (-OCH3)enhances lipophilicity while the glycosidic chains increase hydrophilicity. This amphiphilic nature enables icariin to embed into the mitochondrial membrane, thereby maintaining its structural integrity and stabilizing the mitochondrial membrane potential (Δψm). In addition, icariin upregulates the BDNF/TrkB/CREB signaling pathway, promoting the expression of brain-derived neurotrophic factor (BDNF) and its receptor tropomyosin receptor kinase B (TrkB), as well as enhancing the levels of phosphorylated cAMP response element-binding protein (p-CREB). Through these mechanisms, icariin effectively reverses Aβ25–35-induced neuronal apoptosis, reduces the number of TUNEL-positive cells, and prevents the activation of the apoptotic signaling cascade (Liu et al., 2018). Icariin can effectively inhibit cell apoptosisin AD by restoring the inhibited Wnt/β-catenin signaling pathway, up-regulating the expression of Wnt-3a and promoting the phosphorylation of GSK-3β at Ser-9 (Xiao et al., 2022). In a study on the effect of icariin on the neurobehavioral ability of AD mice and its mechanism, it was found that ferroptosis was activated in AD model mice. Further studies have demonstrated that the hydroxyl groups at the C-5 and C-4′ positions of icariin confer antioxidant capacity, functioning as hydrogen donors to scavenge free radicals, thereby inhibiting mitochondrial lipid peroxidation and iron-driven lipid peroxidation chain reactions to attenuate ferroptosis, ultimately ameliorating cognitive deficits and behavioral abnormalities in mice. In addition, recent evidence has identified MDM2 as a potential key molecular target of icariin, through which it upregulates the expression of SLC7A11 and suppresses neuronal ferroptosis, thereby providing a plausible mechanistic basis for the prevention and treatment of Alzheimer’s disease. (Yang Y. et al., 2023). In addition, icariin can significantly improve neurological dysfunction, effectively prevent D-galactose (D-gal) -induced cell damage and apoptosis, and block the opening of mitochondrial permeability transition pore (mPTP) to improve mitochondrial dysfunction and inhibit excessive mitophagy (Hu S. S. et al., 2024).

#### 5.4.2 Salidroside

Salidroside is a metabolite extracted from the root and stem of the medicinal plant *Rhodiola rosea L [*Crassulaceae*]* It has a variety of pharmacological effects such as anti-fatigue, anti-aging, immune regulation and free radical scavenging. Salidroside possesses a p-hydroxyphenylethanol aglycone as its core skeleton, in which the phenolic hydroxyl group (–OH) functions as a hydrogen atom/electron donor to directly scavenge reactive oxygen species (ROS) and free radicals, thereby inhibiting lipid peroxidation. Moreover, the synergistic contribution of its phenolic hydroxyl group (electron-donating) and glycosidic moiety (–Glc) endows salidroside with amphiphilic properties, enabling it to exert pharmacological effects not only in aqueous environments (e.g.,.cytosol, plasma) but also in membrane-associated environments (e.g., cellular membranes, mitochondrial membranes, lipid rafts). This amphiphilicity facilitates both antioxidant defense and modulation of redox-sensitive signaling pathways, underscoring the critical structure–activity relationship (SAR) underlying its neuroprotective and cytoprotective activities. Xie et al. reported that salidroside exhibits significant potential in attenuating oxidative stress and inflammatory injury in neuronal cells, which is largely attributable to its p-hydroxyphenylethanol aglycone. The phenolic hydroxyl group (–OH) within this scaffold serves as a hydrogen atom/electron donor, directly scavenging ROS and free radicals and thereby suppressing lipid peroxidation, while the attached glucose moiety improves hydrophilicity and bioavailability, enabling systemic distribution. Through these structural features, salidroside enhances intestinal mucosal integrity and reduces circulating granulocyte–macrophage colony-stimulating factor levels, thereby modulating peripheral circulation (Xie et al., 2020). Preliminary studies further demonstrated that salidroside attenuates apoptosis in PC12 cells by inhibiting caspase-3/7 activity and activating ERK1/2 and Akt signaling pathways (Liao et al., 2019). Consistently, salidroside reduces lactate dehydrogenase (LDH) release, lowers malondialdehyde (MDA) and ROS levels, elevates superoxide dismutase (SOD) activity, and stabilizes mitochondrial membrane permeability. Cai et al. investigated its potential in slowing the progression of AD and revealed that the antioxidant capacity conferred by its phenolic hydroxyl groups contributes to the suppression of NLRP3 inflammasome activation and the release of downstream proinflammatory cytokines (Cai et al., 2021). This inhibition of pyroptosis alleviates D-galactose-induced memory deficits and hippocampal neuronal injury by directly targeting the NLRP3/caspase-1 signaling pathway. Moreover, the amphiphilic nature derived from the synergy between the phenolic hydroxyl group and glycosidic moiety allows salidroside to restore primary neuronal morphology, enhance cell viability, decrease LDH release, increase the Bcl-2/Bax ratio, reduce caspase-3 expression, and activate the Nrf2/HO-1 signaling pathway, thereby mitigating neuronal apoptosis (Shuangmei, 2021). Yang provided compelling evidence that the same structural features also underlie its ability to activate the Nrf2/HO-1 pathway, thereby inhibiting ferroptosis in AD model mice and HT22 cells (Yang S. et al., 2022). Subsequent studies further confirmed that salidroside modulates the Nrf2/GPX4 axis, upregulating SLC7A11 and GPX4 expression, which effectively suppresses neuronal ferroptosis (Yang S. et al., 2023). Collectively, these findings underscore that the pharmacological activities of salidroside—including anti-apoptotic, anti-pyroptotic, and anti-ferroptotic effects—are intimately linked to its structural characteristics, providing a mechanistic basis for its neuroprotective role in AD.

## 6 Conclusion

Traditional Chinese medicine natural products come from a wide range of sources. Although these medicinal ingredients are also found in fruits and vegetables, their content is particularly abundant in a variety of plants and herbs. Thousands of years ago, various countries around the world had recognized the therapeutic effects of these natural products through medical practice, including the use of plants and animals in pharmacological and dietary therapies. In recent years, with the continuous deepening of research and the continuous advancement of technology, countries such as China, Korean, Japan, and India, among others, have paid particular attention to traditional Chinese herbal medicines and their shared metabolites, many of which are enriched in TCM, Kampo, and Ayurveda traditions, thus serving as valuable resources for new drug development in a global ethnopharmacological context”. In Korean medicine, the unique Ginsenoside Rg3 of red ginseng and curcumin in Indian Ayurvedic medicine play significant roles in promoting autophagy, clearing Aβ, inhibiting tau phosphorylation, and reducing neuroinflammation and apoptosis. For the use of compound decoction, the ginsenosides and astragalosides in the Buzhongyiqi Decoction (China/Korea) can significantly reduce the expression level of Aβ42 in the cerebral cortex. The standardized extract of Ginkgo biloba (China/EU) can inhibit the phosphorylation of tau protein. The Icariin, 2,3,5,4′-tetrahydroxyldiphenylethylene-2-O-glucoside (TGSH) and Senegenin in the Bushentiansui Decoction (China) can inhibit cell apoptosis and restore memory. Whether derived from traditional Chinese medicine, Korean medicine, or other global traditional medical systems, traditional Chinese herbal medicine and its metabolites are valuable resources for combating Alzheimer’s disease. Although these have complex and multifaceted regulatory mechanisms for various forms of PCD, their potential as therapeutic agents for AD remains indisputable.

Previous studies have clearly indicated that both senile plaques formed by excessive deposition of Aβ and NFTs formed by excessive phosphorylation of Tau protein are closely associated with PCD. Although a large number of experiments have supported this view, most studies have mainly focused on regulating apoptosis and autophagy. In contrast, there are relatively few studies on targeted regulation of other forms of PCD, such as pyroptosis, necroptosis, ferroptosis, cuproptosis, *etc.*, and the crosstalk relationships among various forms of PCD. Therefore, based on this, this paper clarifies the bidirectional pathological relationships between various forms of PCD and AD, and explains the crosstalk relationships among various forms of PCD with autophagy as the core. In addition, this study further clarifies that a certain traditional Chinese medicine natural product has the ability to multi-target and multi-dimensionally regulate various forms of PCD and their related signaling pathways in AD, thereby improving AD, indicating its potential for treating and preventing AD. For example, berberine listed in this paper can improve AD by inhibiting apoptosis, autophagy, and ferroptosis, and salidroside can improve AD by inhibiting apoptosis, pyroptosis, and ferroptosis.

Through research, it has been demonstrated that the efficacy of traditional Chinese medicine natural products in modulating PCD is closely associated with drug concentration. Specifically, berberine suppresses ferroptosis in HT22 cells at 30–60 μM and exhibits optimal anti-apoptotic activity in PC12 cells at 50–200 μM (Hu F. et al., 2024; Huang et al., 2022); salidroside shows the strongest effects at 40 μM in HT22 cells and 50 mg/kgd in Aβ1–42-induced mice; ginsenosides such as Rg1 and Rd are most effective at 5–10 μM, whereas Rb1 demonstrates maximal anti-apoptotic protection at 10 nmol/L compared with weaker effects at both 0.1 nmol/L and 1 μM (Luo, 2024). It is noteworthy that in certain experimental studies, it has been observed that specific traditional Chinese medicine natural products exhibit optimal efficacy at high doses. For instance, in the context of enhancing cell apoptosis, the Bcl-2/Bax ratio was found to be the highest in the high-dose group of tenuigenin (Yang et al., 2013). In addition, the effectiveness of traditional Chinese medicine natural products is not only dose-dependent but may also be influenced by the duration of use. Therefore, during application, it is crucial to avoid excessive dosages or prolonged usage to mitigate the risk of adverse reactions and toxic side effects. Moreover, cuproptosis represents a relatively novel form of PCD. Consequently, the mechanisms by which Plant metabolites regulate PCD remain incompletely understood, and effective therapeutic agents targeting this pathway are scarce. According to current research, cuproptosis is closely associated with elevated ROS levels. Experimental evidence demonstrates that curcumin, quercetin, and epigallocatechin gallate (EGCG) suppress cuproptosis *via* antioxidant pathways (Li et al., 2025; Shen et al., 2018; Sun et al., 2017; Rezai-Zadeh et al., 2008). Thus, we hypothesize that TCM-derived natural metabolites with antioxidant properties and the capacity to inhibit ROS expression may possess potential for inhibiting cuproptosis in cells, thereby contributing to the prevention and treatment of AD.

In conclusion, despite the intricate and multifaceted regulatory mechanisms of traditional Chinese medicine natural products on various forms of PCD, their efficacy as therapeutic agents for AD remains compelling. To ensure scientific rigor, we have summarized the minimum effective concentrations (MECs), representative dose ranges, and optimal concentrations of the major metabolites discussed in this review in a supplementary table, rather than repeating them in each section. This provides a unified methodological basis for all efficacy claims, highlighting that these pharmacological activities are strictly dependent on structure–activity relationships (SAR) and experimentally defined doses (Table 7). By targeting pathways such as apoptosis, autophagy, necroptosis, ferroptosis, and cuproptosis, they can mitigate amyloid-β deposition, reduce neurotoxicity, protect neurons from damage, and thereby alleviate the pathological hallmarks of AD. These findings have been corroborated through animal and cellular experiments. Currently, the mechanistic investigations linking AD to PCD modes like apoptosis, autophagy, and ferroptosis are relatively comprehensive, and the regulatory effects of TCM natural products on these three canonical forms of PCD have been extensively explored. However, the investigation into emerging novel PCD mechanisms, such as cuproptosis, panoptosis, and disulfidoptosis, remains limited. The connections between these mechanisms and AD, as well as the modulatory effects of natural products from traditional Chinese medicine on them, warrant further exploration. Another issue is the different parts of the same traditional Chinese medicine, or the different components of the same traditional Chinese medicine. Comparative studies are needed to determine which type of traditional Chinese medicine is more effective, such as astragalus polysaccharides and astragalus saponins, among different components of the same herb. In the future, we will focus on elucidating the specific role of cuproptosis in the pathogenesis of AD and how natural products from traditional Chinese medicine regulate cuproptosis to prevent and treat this condition. Additionally, in addition to cell programmed death, Alzheimer’s disease is associated with aging, family inheritance, neuroinflammation, vascular factors and even excessive exposure to environmental pollutants and unhealthy lifestyle. We will continue to explore more natural products of Traditional Chinese medicine with potential therapeutic effects, and adopt emerging experimental tools such as single-cell omics tools, *in vivo* biosensor technology, gene editing and microfluidic chip technology to provide more effective options for the prevention and treatment of Alzheimer’s disease with TCM natural products and explore deeper potential effects. By integrating molecular docking and network pharmacology approaches, we aim to enhance treatment efficacy and improve the quality of life for patients with AD.

**TABLE 7:** Representative plant metabolites regulating PCD in AD: pharmacological parameters and mechanisms.

Metabolite	*In vitro* models & mechanisms	Effective concentration range	Optimal concentration	Optimal *in vivo* dosage	Duration	Key pathways and proteins	Reference
Berberine	HT22 (erastin-induced ferroptosis) PC12/N2a (Aβ, apoptosis)	30–200 μM	60 μM (ferroptosis) 50–200 μM (apoptosis)	30–50 mg/kg·d (AD mice)	2–4 weeks	Nrf2–HO-1/GPX4, AMPK, Bcl-2/Bax, caspase-3	Huang et al. (2022)
Sinomenine	PC12 (H_2_O_2_ oxidative stress, apoptosis)	0.1–5 μM	1–2 μM	30–90 mg/kg·d (AD mice)	1–3 weeks	Nrf2,NF-κB, MAPK, caspase-3	Shukla and Sharma (2011)
Matrine	PC12 (Aβ25–35 apoptosis) N2a (oxidative stress)	10–50 μM	20–30 μM	10–40 mg/kg·d (AD mice)	2–6 weeks	ROS,Bcl-2/Bax, caspase-3, PI3K/Akt	Jiang and Wang (2020)
Astragalosides (AS-IV)	SH-SY5Y (6-OHDA-induced apoptosis)	50–200 μM	100 μM	20–80 mg/kg·d (AD mice)	3–6 weeks	JAK2/STAT3 NF-κB, Nrf2/HO-1	Xu et al. (2021)
Tenuigenin (Senegenin)	PC12 (apoptosis, autophagy)	5–50 μM	50 μM	30–60 mg/kg·d (AD mice)	4–8 weeks	Bcl-2/Bax PINK1/Parkin, caspase-3	Chen et al. (2010)
Astragalus polysaccharides (APS)	PC12/HT22 oxidative stress model (H_2_O_2_-induced)	100–1000 μg/mL	250–500 μg/mL	200 mg/kg·d (AD mice); 500 mg/kg·d (AD mice)	200 mg/kg·d (2 months) 500 mg/kg·d (7 weeks)	Nrf2/Keap1, SOD, GSH-Px, MDA, JAK/STAT, Toll/IMD, PI3K/Akt/mTOR	Huang et al. (2017)
Lycium barbarum polysaccharides (LBP)	SH-SY5Y (Aβ toxicity, apoptosis)	100–400 μg/mL	200–400 μg/mL	10–40 mg/kg·d (AD mice)	3–8 weeks	PI3K/Akt, Bcl-2, Bax, caspase-3, mitophagy	Zhao et al. (2016)
Icariin	SH-SY5Y, PC12 (Aβ-induced apoptosis)	5–20 μM	10 μM	30–60 mg/kg·d (AD mice)	3–6 weeks	BDNF/TrkB/CREB, caspase-3, LC3-II	Fu et al. (2018)
Salidroside	HT22 (oxidative stress, ferroptosis) PC12 (apoptosis, pyroptosis)	20–80 μM	40 μM	50 mg/kg·d (AD mice)	2–4 weeks	Nrf2/HO-1, GPX4, NLRP3/caspase-1, Bcl-2/Bax	Yang et al. (2022c)
Huperzine A	Neurons (AChE inhibition, apoptosis regulation)	nM level effective	0.1–1 μM	0.2–0.4 mg/day (humans) 0.2–0.7 mg/kg (mice)	weeks–months	AChE inhibition, Bcl-2, Bax, caspase-3	Yang et al. (2013)
